# Hypoxia-inducible factor cell non-autonomously regulates *C. elegans* stress responses and behavior via a nuclear receptor

**DOI:** 10.7554/eLife.36828

**Published:** 2018-07-16

**Authors:** Corinne L Pender, H Robert Horvitz

**Affiliations:** 1 Department of Biology, Howard Hughes Medical InstituteMassachusetts Institute of TechnologyCambridgeUnited States; 2 McGovern Institute for Brain ResearchMassachusetts Institute of TechnologyCambridgeUnited States; 3 Koch Institute for Integrative Cancer ResearchMassachusetts Institute of TechnologyCambridgeUnited States; University of WashingtonUnited States; Max Planck Institute for Heart and Lung ResearchGermany

**Keywords:** hypoxia-inducible factor, cytochrome P450, nuclear hormone receptor, *C. elegans*

## Abstract

The HIF (hypoxia-inducible factor) transcription factor is the master regulator of the metazoan response to chronic hypoxia. In addition to promoting adaptations to low oxygen, HIF drives cytoprotective mechanisms in response to stresses and modulates neural circuit function. How most HIF targets act in the control of the diverse aspects of HIF-regulated biology remains unknown. We discovered that a HIF target, the *C. elegans* gene *cyp-36A1*, is required for numerous HIF-dependent processes, including modulation of gene expression, stress resistance, and behavior. *cyp-36A1* encodes a cytochrome P450 enzyme that we show controls expression of more than a third of HIF-induced genes. CYP-36A1 acts cell non-autonomously by regulating the activity of the nuclear hormone receptor NHR-46, suggesting that CYP-36A1 functions as a biosynthetic enzyme for a hormone ligand of this receptor. We propose that regulation of HIF effectors through activation of cytochrome P450 enzyme/nuclear receptor signaling pathways could similarly occur in humans.

## Introduction

The capacity to sense and respond to oxygen deprivation, or hypoxia, is crucial to normal physiological function and survival of aerobic organisms, which require oxygen to perform respiration and generate energy in the form of ATP. The fundamental importance of a mechanism to detect and react to low oxygen is reflected in the presence of a conserved hypoxia-response pathway in most animal cells. This pathway consists of the transcription factor HIF, or hypoxia-inducible factor, and its negative regulator, the prolyl hydroxylase EGLN, which together mediate a diversity of metabolic and physiological adaptations to hypoxia. The three human EGLNs, which were identified as homologs of the *C. elegans* protein EGL-9, function as oxygen sensors. In the presence of oxygen, EGLN hydroxylates the HIF α-subunit (HIFα), allowing the von Hippel-Lindau (VHL) E3 ubiquitin ligase to promote HIFα degradation (Maxwell et al., 1999; Jaakkola et al., 2001; Epstein et al., 2001; Ivan et al., 2001). In conditions of low oxygen, HIFα is stabilized and acts with its partner HIFβ to drive adaptations to hypoxia through activation of its transcriptional targets (Kaelin and Ratcliffe, 2008; Semenza, 2011; Wang et al., 1995).

The canonical function of the EGLN/HIF pathway is to regulate genes that either increase oxygen availability, for example by promoting erythropoiesis and angiogenesis, or reduce the cellular requirement for oxygen, for example by driving a shift from oxidative phosphorylation to glycolytic metabolism. However, a growing body of work has found roles for the EGLN/HIF pathway in controlling other aspects of animal physiology and behavior. HIF promotes the response to numerous stressors, including infection, proteotoxicity, and oxidative stress (Palazon et al., 2014; Schito and Rey, 2018; Nakazawa et al., 2016; Powell-Coffman, 2010). HIF activation is associated with increased lifespan in *C. elegans* (Mehta et al., 2009; Zhang et al., 2009; Chen et al., 2009; Lee et al., 2010); this longevity phenotype likely stems from the improved stress resistance associated with HIF activity, as is often the case for pathways regulating longevity (Leiser et al., 2015; Shore and Ruvkun, 2013). The EGLN/HIF pathway also modulates several behaviors of *C. elegans* following prolonged hypoxia exposure, suggesting a role for this pathway in tuning neural circuit function (Chang and Bargmann, 2008; Pocock and Hobert, 2010; Ma et al., 2012). The mechanisms by which HIF mediates these non-canonical physiological and behavioral changes remain poorly defined.

Here we report the discovery of an endocrine signaling pathway that regulates multiple aspects of physiology and behavior downstream of HIF in *C. elegans*. From a genetic screen for suppressors of an *egl-9(lf)* mutant behavioral defect, we identified a cytochrome P450 gene, *cyp-36A1*, that is required for modulation of egg-laying behavior by the *egl-9*/*hif-1* pathway. *cyp-36A1* is transcriptionally upregulated in hypoxia or *egl-9(lf)* mutants, in which HIF-1 is constitutively active, and appears to be a direct HIF-1 target. *cyp-36A1* controls expression of more than a third of HIF-1-upregulated genes, demonstrating that *cyp-36A1* acts broadly downstream of *hif-1*. Regulation of gene expression and behavior by *cyp-36A1* occurs cell non-autonomously, and the downstream effector of *cyp-36A1* is the nuclear hormone receptor *nhr-46*, indicating that the likely function of CYP-36A1 is to generate a diffusible signal that controls NHR-46 activity. In addition to modulating behavior and gene expression, *cyp-36A1* and *nhr-46* mediate multiple forms of stress resistance associated with HIF activation. We conclude that CYP-36A1 and NHR-46 are important downstream effectors of the EGL-9/HIF pathway and function together to regulate a wide range of HIF-mediated physiology.

## Results

### A screen for suppressors of the *egl-9(lf)* egg-laying defect identifies the cytochrome P450 gene *cyp-36A1*

To identify novel, functionally important HIF effectors, we analyzed the modulation of *C. elegans* egg laying, the behavior that led our laboratory to discover the first EGLN gene, *egl-9*, and the first known functional role for any member of the EGLN/HIF pathway (Trent et al., 1983). *egl-9(lf)* mutants, in which HIF-1 is constitutively active, are defective in egg laying and become bloated with eggs as adults. Although the egg-laying defect of *egl-9(lf)* mutants is well-established, the downstream effectors of EGL-9 and HIF-1 in regulating egg-laying behavior remain unknown.

**Figure 1.**
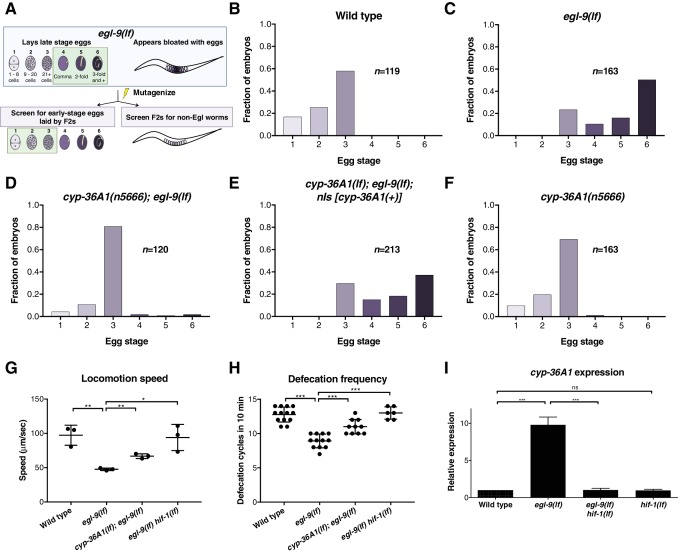
The cytochrome P450 gene *cyp-36A1* is an effector of the hypoxia-response pathway that regulates behavior. (**A**) Schematic of the screen design. Stages of embryonic development adapted from Ringstad and Horvitz (2008) and Paquin et al. (2016). (**B–F**) Distribution of stages of eggs newly laid by adult hermaphrodites, used as a proxy for egg retention time *in utero*, of animals of the indicated genotypes. (**B**) Stages of eggs laid by wild-type animals. (**C**) *egl-9* loss-of-function (*lf*) mutants laid later stage eggs than the wild type (p<0.001, Chi-square test with Holm-Bonferroni correction). (**D**) *cyp-36A1(n5666)* suppressed the egg-laying defect of *egl-9(lf)* mutants (p<0.001). (**E**) The *cyp-36A1(+)* transgene, which contains wild-type *cyp-36A1*, rescued the suppression of the egg-laying defect observed in *cyp-36A1(n5666); egl-9(lf)* mutants (p<0.001). (**F**) *cyp-36A1(n5666)* mutants displayed wild-type egg laying (p>0.05). (**G**) *egl-9(lf)* mutants were defective in locomotion rate, and this defect was suppressed by *hif-1(lf)* and *cyp-36A1(lf)* mutations. Mean ± SD of *n* = 3 biological replicates, *p<0.05, **p<0.01 considered significant (Student’s t-test with Holm-Bonferroni correction). (**H**) *egl-9(lf)* mutants were defective in defecation rate, and this defect was suppressed by *hif-1(lf)* and *cyp-36A1(lf).* Mean ± SD of *n* ≥ 6 animals, ***p<0.001 considered significant (Student’s t-test with Holm-Bonferroni correction). (**I**) Relative expression of *cyp-36A1* mRNA in the wild type, *egl-9(lf)*, *egl-9(lf) hif-1(lf)*, and *hif-1(lf)* mutants, measured by qRT-PCR and normalized to the expression of the large ribosomal subunit *rpl-32*. Mean ± SD of *n* = 3 biological replicates, ***p<0.001 considered significant. ns, not significant (Student’s t-test with Holm-Bonferroni correction). Alleles used for (**B–F**) were *cyp-36A1(n5666)*, *egl-9(n586)*, and *nIs674* (*nIs* [*cyp-36A1(+)*]), and all strains used in (**B–F**) contained the *nIs470* (*P_cysl-2_::gfp*) transgene. Alleles used for (**G–I**) were *egl-9(sa307), hif-1(ia4)*, and *cyp-36A1(gk824636).*

We performed a mutagenesis screen to identify genes that act in response to *egl-9* to control egg laying. Specifically, we screened for second-site mutations that suppressed the egg-laying defect of *egl-9(lf)* animals (Figure 1A). Such suppressors could define genes that function downstream of *egl-9.* Two isolates from this screen were allelic to *hif-1* (Figure 1—figure supplement 1A and B), consistent with a previous observation that *hif-1(lf)* suppresses the *egl-9(lf)* egg-laying defect (Bishop et al., 2004) and validating the screen as a means of identifying components of the HIF-1 pathway. A third isolate, *n5666*, was not allelic to *hif-1* and had a G106R missense mutation in the gene *cyp-36A1*, which encodes a cytochrome P450 enzyme (Figure 1—figure supplement 1A and C). A transgene carrying a wild-type copy of *cyp-36A1* fully rescued the suppression by *n5666* of the *egl-9(lf)* egg-laying defect, demonstrating that the mutation in *cyp-36A1* is the causative mutation and suggesting that the suppression phenotype is caused by reduction of *cyp-36A1* function (Figure 1B–1E). Confirming these conclusions, a nonsense allele of *cyp-36A1* also suppressed the *egl-9(lf)* egg-laying defect (Figure 3—figure supplement 1B and C). *cyp-36A1(lf)* single mutants did not exhibit hyperactive egg-laying behavior (Figure 1F and Figure 3—figure supplement 1A and D), indicating that suppression of the *egl-9(lf)* egg-laying defect by *cyp-36A1(lf)* is not a consequence of a nonspecific increase in egg-laying rate. We further showed that *cyp-36A1(lf)* suppressed the previously reported egg-laying defect of hypoxia-exposed worms (Miller and Roth, 2009), demonstrating a role for CYP-36A1 under physiological conditions of HIF-1 activation (Figure 1—figure supplement 2). We then analyzed the role of *cyp-36A1* in regulating other behaviors. We observed that *egl-9(lf)* mutants have reduced locomotion and defecation rates, both of which were suppressed by *hif-1(lf)* (Figure 1G and H). *cyp-36A1(lf)* partially suppressed the slow locomotion and defecation rates of *egl-9(lf)* mutants, showing that CYP-36A1 modulates not only egg laying but also multiple other HIF-1-regulated behaviors.

**Figure 1—figure supplement 1.**
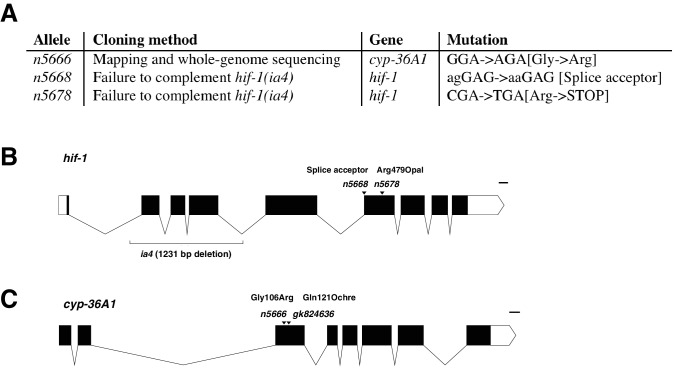
A screen for suppressors of the *egl-9(lf)* egg-laying defect. (**A**) Summary of three cloned alleles from a suppressor screen of the *egl-9(lf)* egg-defect. (**B**) Gene diagram of *hif-1*; isoform *F38A6.3b* is shown. The *ia4* deletion allele was generated by deletion screening (Jiang et al., 2001). Scale bar, 100 bases. (**C**) Gene diagram of *cyp-36A1*. The *gk824636* allele is from the Million Mutation Project (Thompson et al., 2013). Scale bar, 100 bases.

**Figure 1—figure supplement 2.**
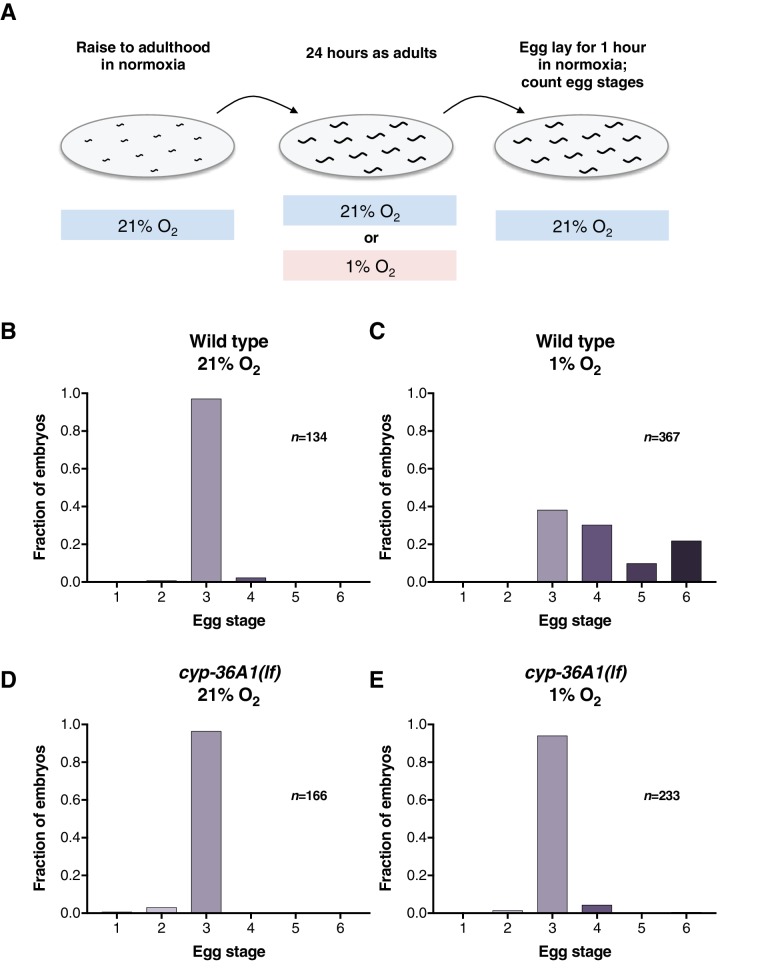
*cyp-36A1(lf)* suppresses the egg-laying defect of hypoxia-exposed animals. (**A**) Schematic depicting experimental design. (**B–E**) Distribution of stages of eggs laid by adult hermaphrodites. (**B**) Stages of eggs laid by wild-type animals grown in normoxia (21% O_2_). (**C**) Animals exposed to hypoxia (1% O_2_) for 24 hr as adults laid later stage eggs than those in normoxia (p<0.001, Chi-square test with Holm-Bonferroni correction). (**D**) *cyp-36A1(gk824636)* mutants grown in normoxia displayed wild-type egg laying (p>0.05). (**E**) *cyp-36A1(gk824636)* mutants exposed to 1% O_2_ laid eggs at earlier stages than wild-type animals in the same condition (p<0.001).

Next we observed that *cyp-36A1* expression is increased in *egl-9(lf)* mutants in a *hif-1*-dependent manner (Figure 1I), consistent with results from an earlier genome-wide microarray study that identified *cyp-36A1* as one of 63 genes regulated by *hif-1* in hypoxia-exposed worms (Shen et al., 2005). ChIP-seq of HIF-1 by the modERN project showed HIF-1 binding at two sites near the *cyp-36A1* coding region, one 5’ to the start of the gene and one in the first intron (Kudron et al., 2018); both of these sites contain the HIF binding motif 5’RCGTG (Kaelin and Ratcliffe, 2008). We conclude that *cyp-36A1* is a downstream effector of the hypoxia-response pathway that regulates multiple behaviors and that *cyp-36A1* likely is a direct transcriptional target of HIF-1.

### CYP-36A1 regulates gene expression changes and stress resistance downstream of HIF-1

**Figure 2.**
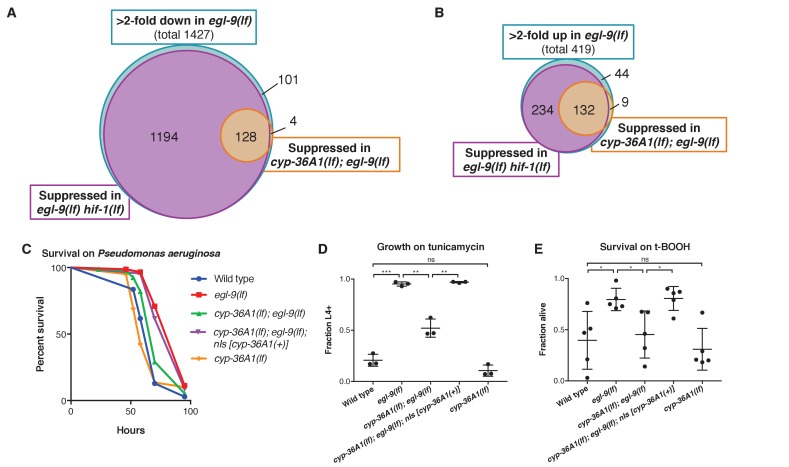
CYP-36A1 acts downstream of HIF-1 to regulate gene expression changes and stress responses. (**A**) Blue circle: Genes that were at least twofold downregulated in *egl-9(lf)* mutants. Purple and orange circles: Subset of *egl-9(lf)*-downregulated genes that were significantly upregulated in *egl-9(lf) hif-1(lf)* double mutants (purple) or *cyp-36A1(lf); egl-9(lf)* double mutants (orange) vs. *egl-9(lf)* single mutants. (**B**) Blue circle: Genes that were at least twofold upregulated in *egl-9(lf)* mutants. Purple and orange circles: Subset of *egl-9(lf)*-upregulated genes that were significantly downregulated in *egl-9(lf) hif-1(lf)* double mutants (purple) or *cyp-36A1(lf); egl-9(lf)* double mutants (orange) vs. *egl-9(lf)* single mutants. Significance for all comparisons in (**A**) and (**B**) was based on two biological replicates and determined by the Benjamini-Hochberg procedure with a false-discovery rate of 0.05. (**C**) Survival of animals grown from the L4 larval stage on the pathogen *Pseudomonas aeruginosa*. Wild type (*n* = 86 animals) vs. *egl-9(lf)* (*n* = 154) p<0.001; *egl-9(lf)* vs. *cyp-36A1(lf); egl-9(lf)* (*n* = 83), p<0.001; *cyp-36A1(lf); egl-9(lf)* vs. *cyp-36A1(lf); egl-9(lf); nIs [cyp-36A1(+)]* (*n* = 97), p<0.001; wild type vs. *cyp-36A1(lf)* (*n* = 61) p<0.05, as determined by the log-rank (Mantel-Cox) test, correcting for multiple comparisons with the Holm-Bonferroni method. See figure supplement for replicate data. (**D**) Survival of animals to the L4 larval stage or later after growth for three days from the L1 larval stage on plates containing 5 μg/ml tunicamycin. Mean ± SD of *n* = 3 biological replicates. **p<0.01, ***p<0.001 considered significant. ns (p>0.05), not significant (Student’s t-test with Holm-Bonferroni correction). (**E**) Survival of animals exposed to 7.5 mM tert-butyl hydroperoxide for 10 hr as young adults. Mean ± SD of *n* = 5 biological replicates. *p<0.05 considered significant. ns (p>0.05), not significant (Student’s t-test with Holm-Bonferroni correction). Alleles used for (**A**) and (**B**) were *egl-9(sa307), hif-1(ia4),* and *cyp-36A1(gk824636).* Alleles used for (**C–E**) were *egl-9(n586)*, *cyp-36A1(n5666)*, and *nIs674* (*nIs [cyp-36A1(+)]*), and all strains used in (**C–E**) contained the *nIs470* (*P_cysl-2_::gfp*) transgene. 10.7554/eLife.36828.007Figure 2—source data 1.RNA-seq GO enrichment analysis.Gene ontology enrichment analysis terms for genes more than twofold upregulated in *egl-9* mutants and significantly suppressed by *hif-1(lf)* and *cyp-36A1(lf)*.

We sought to determine if CYP-36A1 regulates other HIF-1-dependent processes. Based on sequence identity, CYP-36A1 is most closely related to the CYP2 family of cytochrome P450 enzymes, which function in both detoxification of xenobiotics and metabolism of endogenous molecules (Nebert et al., 2013). CYP2 family members and other CYPs can act on endogenous substrates to generate diffusible signaling molecules that regulate gene expression, such as eicosanoids and steroid hormones (Rendic and Guengerich, 2015; Dennis and Norris, 2015; Evans and Mangelsdorf, 2014). We hypothesized that CYP-36A1 might function in a transcriptional cascade to mediate aspects of HIF-1-dependent gene regulation. We performed an RNA-seq experiment comparing the wild type, *egl-9(lf)* mutants, *egl-9(lf) hif-1(lf)* double mutants, and *cyp-36A1(lf); egl-9(lf)* double mutants. We found that *hif-1(lf)* suppressed *egl-9*-dependent gene expression for 93% of *egl-9(lf)*-downregulated genes and 87% of *egl-9(lf)*-upregulated genes, indicating that most but not all regulation of transcription by *egl-9* occurs through *hif-1*, consistent with previous work (Angeles-Albores et al., 2018). We further found that 36% of HIF-1-upregulated genes (i.e. genes that are upregulated in *egl-9(lf)* mutants and suppressed by *hif-1(lf)*) and 10% of HIF-1-downregulated genes were also regulated by *cyp-36A1* (Figure 2A and B and Supplementary files 1 and 2). We focused on the HIF-1-upregulated genes, for which CYP-36A1 function appeared to be more broadly required. Gene ontology (GO) enrichment analysis of these HIF-1/CYP-36A1-upregulated genes suggested a role for *cyp-36A1* in regulating stress resistance downstream of *egl-9* and *hif-1* (Figure 2—source data 1). The EGL-9/HIF-1 pathway has previously been implicated in responses to numerous stressors in both nematodes and mammals, with crosstalk occurring between HIF and regulators of the immune response, unfolded protein response, and other stress-response pathways (Palazon et al., 2014; Schito and Rey, 2018; Wouters and Koritzinsky, 2008; Nakazawa et al., 2016; Powell-Coffman, 2010). We tested whether CYP-36A1 is involved in the response to three stressors for which HIF-1 is known to mediate resistance in *C. elegans*: infection by the pathogenic bacteria *Pseudomonas aeruginosa* (Darby et al., 1999; Bellier et al., 2009; Shao et al., 2010; Budde and Roth, 2011; Kirienko et al., 2013), tunicamycin-induced ER stress (Leiser et al., 2015), and oxidative stress from tert-butyl hydroperoxide (Bellier et al., 2009). Animals in which HIF-1 is constitutively active because of mutation in *egl-9* or the *C. elegans* VHL homolog *vhl-1* are resistant to these stressors relative to wild-type animals: such mutants survive longer when grown on *Pseudomonas aeruginosa* strain PA14 (Bellier et al., 2009), display reduced tunicamycin-induced growth inhibition (Leiser et al., 2015), and survive exposure to tert-butyl hydroperoxide at a higher rate than the wild type (Bellier et al., 2009). We found that *cyp-36A1(lf); egl-9(lf)* double mutants are more sensitive than *egl-9(lf)* mutants to all three of these stressors (Figure 2C–2E and Figure 2—figure supplement 1), suggesting that CYP-36A1 mediates responses to these stressors downstream of HIF-1. Together the CYP-36A1-dependent changes in gene expression and stress resistance indicate that CYP-36A1 plays a major role in regulating HIF-1-mediated physiology.

**Figure 2—figure supplement 1.**
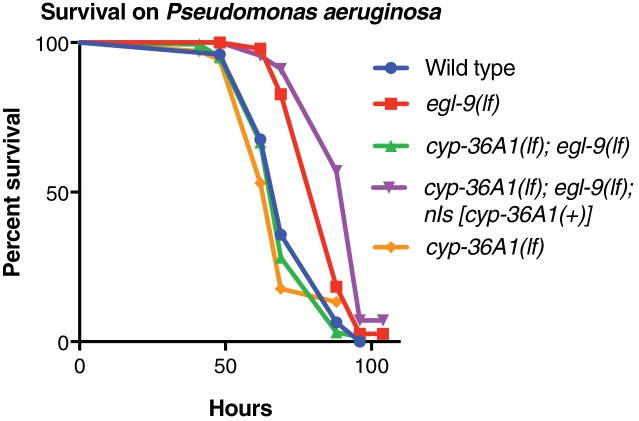
Replicate data for survival on *Pseudomonas aeruginosa*. Replicate data for Figure 2C. Wild type (*n* = 150) vs. *egl-9(lf)* (*n* = 50), p<0.001; *egl-9(lf)* vs. *cyp-36A1(lf); egl-9(lf)* (*n* = 163), p<0.001; *cyp-36A1(lf); egl-9(lf)* vs. *cyp-36A1(lf); egl-9(lf); nIs [cyp-36A1(+)]* (*n* = 51), p<0.001; wild type vs. *cyp-36A1(lf)* (*n* = 35), p>0.05, as determined by the log-rank (Mantel-Cox) test, correcting for multiple comparisons with the Holm-Bonferroni method.

### CYP-36A1 functions cell non-autonomously to regulate gene expression

**Figure 3.**
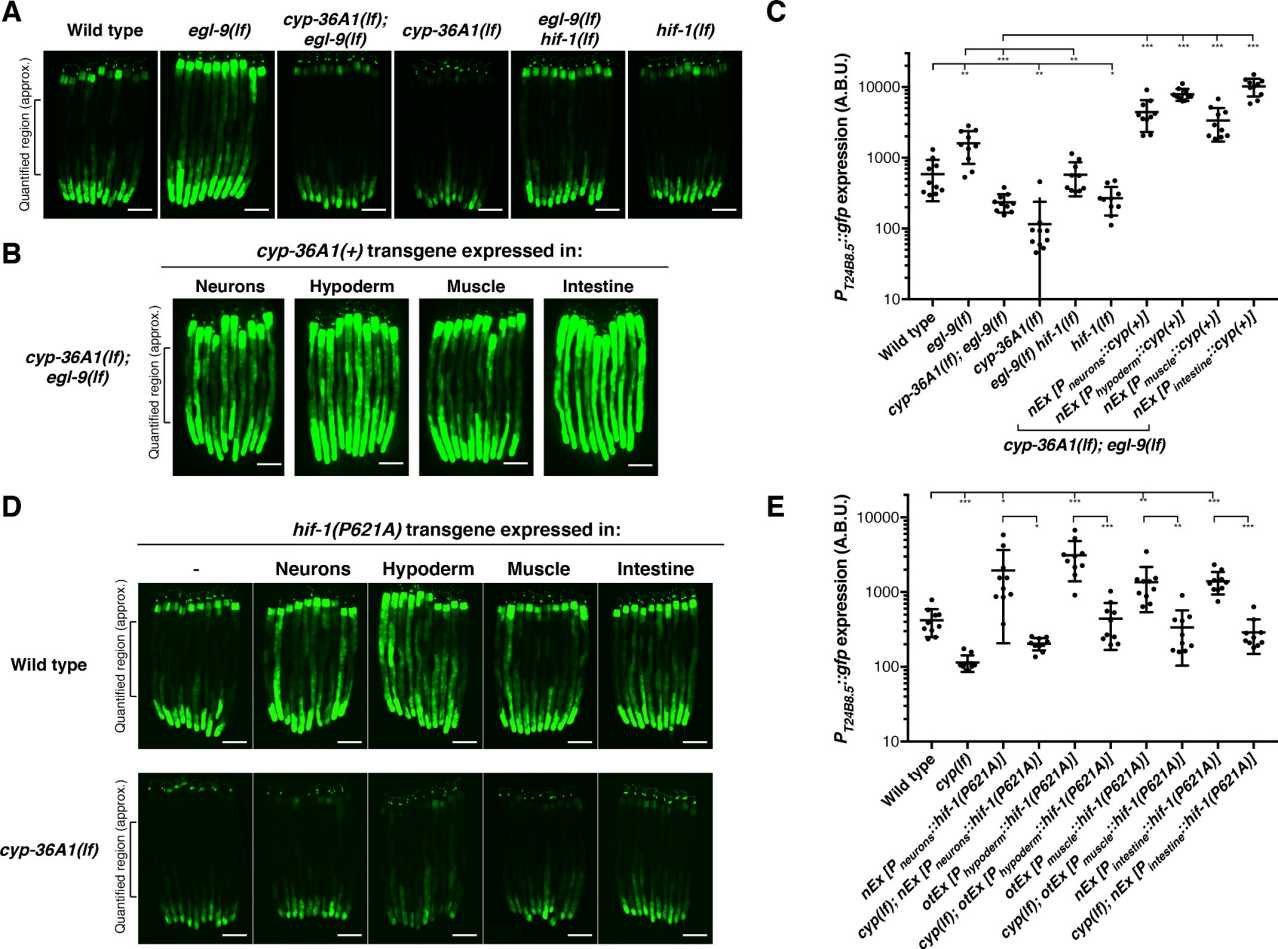
HIF-1 and CYP-36A1 cell non-autonomously regulate expression of a stress-responsive gene. (**A**) *P_T24B8.5_::gfp* expression of the indicated genotypes (*n* = 10 animals per image). Scale bars, 100 μm. (**B**) Expression of *cyp-36A1(+)* specifically in neurons, hypoderm, muscle, or intestine of *cyp-36A1(lf); egl-9(lf)* animals increased expression of *P_T24B8.5_::gfp* in intestine (*n* = 10 animals per image). Scale bars, 100 μm. (**C**) Quantification of fluorescence intensity for (**A**) and (**B**), measured as average intensity for a 300 μm section of the intestine in the midbody of each animal, as indicated. *p<0.05, **p<0.01, ***p<0.001 considered significant (Student’s t-test with Holm-Bonferroni correction). Mean ± SD of *n* = 10 animals. See figure supplement for replicate data. (**D**) Expression of *hif-1(P621A)*, which encodes a stable variant of HIF-1 (Pocock and Hobert, 2008), specifically in neurons, hypoderm, muscle, or intestine increased expression of *P_T24B8.5_::gfp* in intestine; increased expression was suppressed by *cyp-36A1(lf)* (*n* = 10 animals per image). Scale bars, 100 μm. (**E**) Quantification of fluorescence intensity for (**D**), measured as average intensity for a 300 μm section of the intestine in the midbody of each animal, as indicated. *p<0.05, ***p<0.01, ***p<0.001 considered significant (Student’s t-test with Holm-Bonferroni correction). Mean ± SD of *n* = 10 animals. See figure supplement for replicate data. Alleles used were *egl-9(sa307), hif-1(ia4), cyp-36A1(gk824636), nEx2699[P_neurons_::hif-1(P621A)], otEx3156 [P_hypoderm_::hif-1(P621A)]*, *otEx3165 [P_muscle_::hif-1(P621A)]*, *nEx2860 [P_intestine_:: hif-1(P621A)], nEx2853 [P_neurons_::cyp(+)], nEx2856 [P_hypoderm_::cyp(+)], nEx2859 [P_muscle_::cyp(+)],* and *nEx2849 [P_intestine_::cyp(+)].* All strains contained the *agIs219* (*P_T24B8.5_::gfp*) transgene.

We next sought to identify the site of action of CYP-36A1. We hypothesized that CYP-36A1 might function cell non-autonomously, as is the case for other cytochrome P450 enzymes that generate signaling molecules (Nebert et al., 2013; Evans and Mangelsdorf, 2014; Gerisch and Antebi, 2004). We observed *cyp-36A1* expression in many tissues, including neurons, intestine, hypoderm, and muscle (Figure 3—figure supplement 2). To test the hypothesis of cell non-autonomous CYP-36A1 function, we focused on a *cyp-36A1*-mediated abnormality of *egl-9(lf)* mutants for which the site of dysfunction is well defined. Specifically, we examined expression of a GFP transcriptional reporter for the gene *T24B8.5* (Shivers et al., 2009), which is expressed in only the intestine and based on our RNA-seq data is upregulated in *egl-9(lf)* mutants in a *cyp-36A1*-dependent manner. Interestingly, *T24B8.5* expression is also upregulated in response to infection, ER stress, and oxidative stress (Shivers et al., 2009; Lim et al., 2014; Park et al., 2009). Expression of the reporter recapitulated the *T24B8.5* expression changes observed by RNA-seq: increased expression of GFP was observed in *egl-9(lf)* mutants, which was suppressed by a second mutation in either *hif-1* or *cyp-36A1* (Figure 3A and C and Figure 3—figure supplement 3). To determine the site of action of *cyp-36A1* for regulation of intestinal *T24B8.5* expression, we expressed wild-type *cyp-36A1* cDNA using tissue-specific promoters. We found that the low *P_T24B8.5_::gfp* expression of *cyp-36A1(lf); egl-9(lf)* double mutants was rescued by expressing *cyp-36A1(+)* either cell autonomously in the intestine or cell non-autonomously in neurons, hypoderm or body-wall muscle (Figure 3B and C and Figure 3—figure supplement 3). *cyp-36A1(+)* expression in all four tissues also rescued the suppression of the egg-laying defect of *cyp-36A1(lf); egl-9(lf)* mutants (Figure 3—figure supplement 1). Next we found that expressing a nondegradable constitutively active HIF-1 mutant protein (P621A) (Pocock and Hobert, 2008) in any of the same four tissues also promoted intestinal expression of the GFP reporter and that this HIF-1-mediated increase in expression required *cyp-36A1* (Figure 3D and E and Figure 3—figure supplement 4). Thus, CYP-36A1 can function cell non-autonomously to regulate gene expression downstream of HIF-1, consistent with the hypothesis that CYP-36A1 acts by generating a diffusible signal.

**Figure 3—figure supplement 1.**
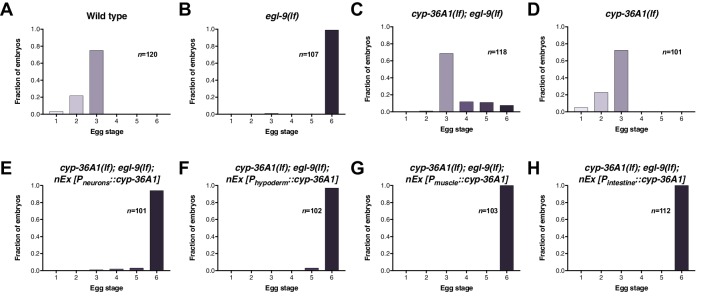
*cyp-36A1* expression in multiple tissues rescues the egg-laying phenotype of *cyp-36A1(lf); egl-9(lf)*. (**A–H**) Distribution of stages of eggs laid by adult hermaphrodites of the indicated genotypes. All strains contained the *agIs219* (*P_T24B8.5_::gfp*) transgene. (**A**) Stages of eggs laid by wild-type animals. (**B**) *egl-9(sa307)* animals laid later stage eggs than wild type (p<0.001, Chi-square test with Holm-Bonferroni correction). (**C**) *cyp-36A1(gk824636)* suppressed the egg-laying defect of *egl-9(sa307)* mutants (p<0.001). (**D**) Stages of eggs laid by *cyp-36A1(gk824636)* single mutants were not significantly different from those laid by wild-type animals (p>0.05). (**E–H**) Expression of *cyp-36A1(+)* specifically in neurons, hypoderm, muscle, or intestine of *cyp-36A1(gk824636); egl-9(sa307)* animals all rescued the suppression by *cyp-36A1(lf)* of the egg-laying defect of *egl-9(lf)* (p<0.001).

**Figure 3—figure supplement 2.**
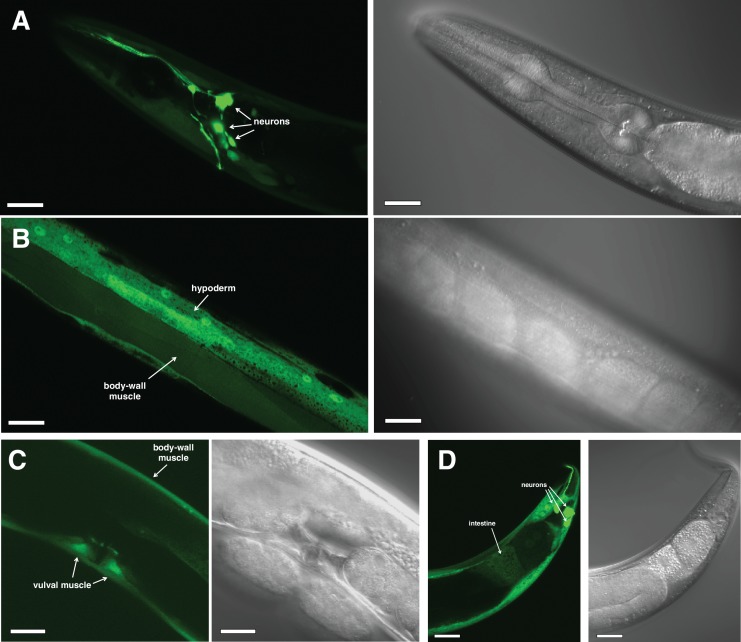
*cyp-36A1* is expressed in many tissues. (**A–D**) Paired fluorescent (left in each panel) and Nomarski (right in each panel) micrographs showing expression of a transcriptional *P_cyp-36A1_::gfp* reporter (*nIs682*) in an adult worm. Expression was observed in the head (**A**), midbody (**B and C**), and tail (**D**), including in neurons, body-wall muscle, vulval muscle, intestine, and hypoderm, as indicated. Scale bars, 20 μm.

**Figure 3—figure supplement 3.**
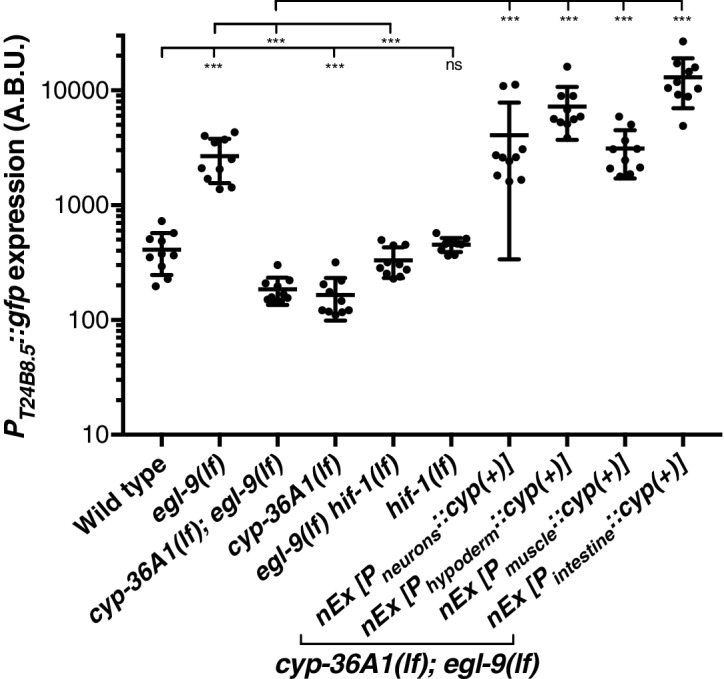
Replicate data for *P_T24B8.5_::gfp* reporter expression. Replicate data for Figure 3C. Quantification of *n* = 10 animals. ***p<0.001 considered significant. ns (p>0.05), not significant (Student’s t-test with Holm-Bonferroni correction).

**Figure 3—figure supplement 4.**
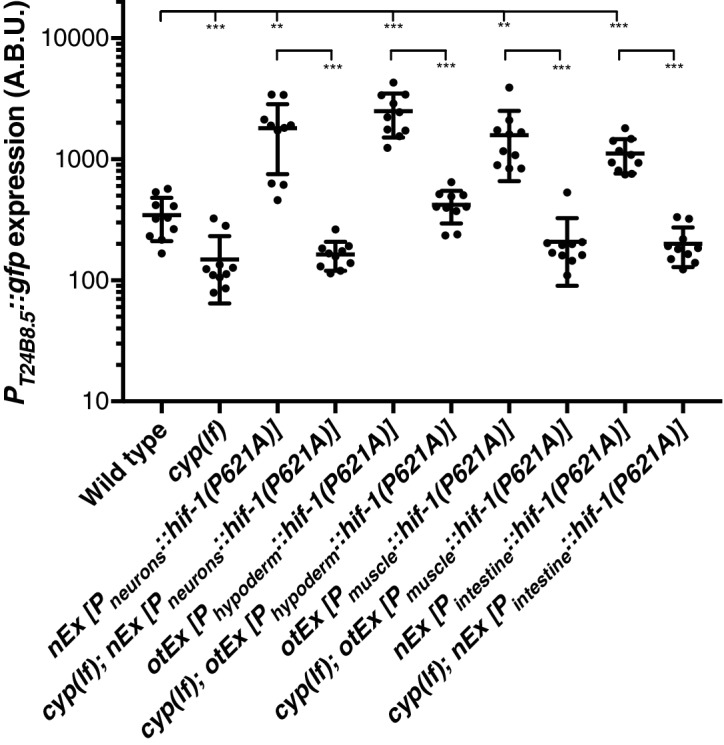
Replicate data for *P_T24B8.5_::gfp* reporter expression. Replicate data for Figure 3E. Quantification of *n* = 10 animals. **p<0.01, ***p<0.001 considered significant (Student’s t-test with Holm-Bonferroni correction).

### A screen for suppressors of *cyp-36A1(lf)* identifies the nuclear receptor gene *nhr-46*

**Figure 4.**
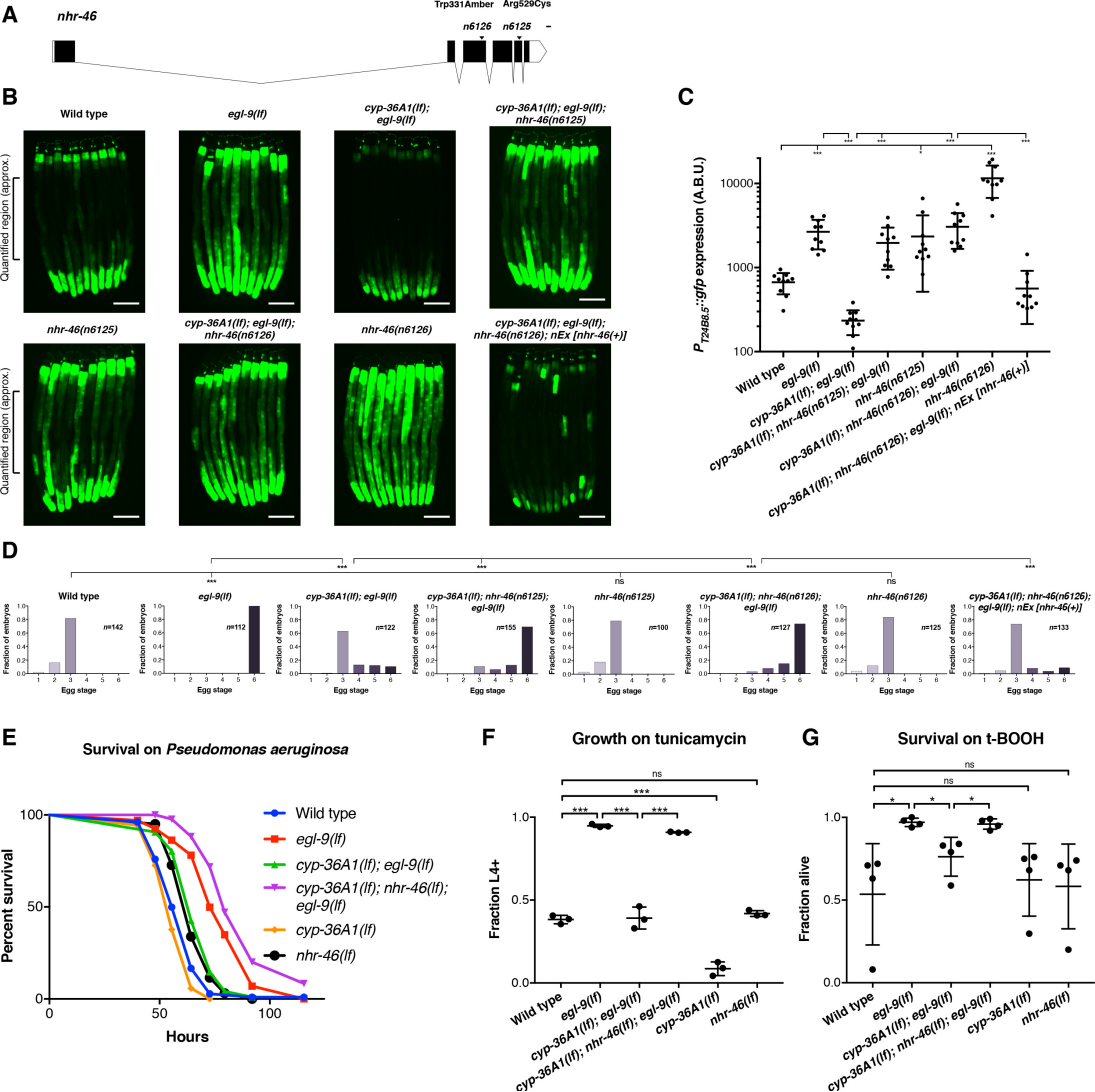
The nuclear hormone receptor NHR-46 acts downstream of CYP-36A1. (**A**) *nhr-46* gene diagram; isoform *C45E5.6b* is shown. *n6125* and *n6126* are in the NHR-46 ligand-binding domain. Scale bar, 100 bases. (**B**) *P_T24B8.5_::gfp* fluorescence of the indicated genotypes (*n* = 10 animals per image). Scale bars, 100 μm. (**C**) Quantification of fluorescence intensity for (**B**), measured as average intensity for a 300 μm section of the intestine in the midbody of each animal, as indicated. *p<0.05, ***p<0.001 considered significant (Student’s t-test with Holm-Bonferroni correction). Mean ±SD of *n* = 10 animals. See figure supplement for replicate data. (**D**) Distribution of stages of eggs newly laid by adult hermaphrodites of the indicated genotypes. ***p<0.001 considered significant. ns (p>0.05), not significant (Chi-square test with Holm-Bonferroni correction). (**E**) Survival of animals grown from the L4 larval stage on the pathogen *Pseudomonas aeruginosa*. Wild type (*n* = 129) vs. *egl-9(lf)* (*n* = 67) p<0.001; *egl-9(lf)* vs. *cyp-36A1(lf); egl-9(lf)* (*n* = 129), p<0.001; *cyp-36A1(lf); egl-9(lf)* vs. *cyp-36A1(lf); nhr-46(lf); egl-9(lf)* (n = 94), p<0.001; wild type vs. *cyp-36A1(lf)* (*n* = 106), p<0.05; wild type vs. *nhr-46(lf)* (*n* = 121), p<0.001, as determined by the log-rank (Mantel-Cox) test, correcting for multiple comparisons with the Holm-Bonferroni method. *egl-9(lf)* allele was *egl-9(n586).* See figure supplement for replicate data. (**F**) Survival of animals to the L4 larval stage or later after growth for three days from the L1 larval stage on plates containing 5 μg/ml tunicamycin. Mean ± SD of *n* = 3 biological replicates. ***p<0.001 considered significant. ns (p>0.05), not significant (Student’s t-test with Holm-Bonferroni correction). (**G**) Survival of animals exposed to 7.5 mM tert-butyl hydroperoxide for 10 hr as young adults. Mean ± SD of *n* = 4 biological replicates. *p<0.05 considered significant. ns, not significant (Student’s t-test with Holm-Bonferroni correction). Alleles used for (**B–G**) were *egl-9(sa307), cyp-36A1(gk824636)*, *nhr-46(n6126)*, and *nEx2586* (*nEx [nhr-46(+)]*) except where otherwise noted. All strains in (**B**), (**C**), (**D**), (**F**), and (**G**) contained the *agIs219* (*P_T24B8.5_::gfp*) transgene.

We performed a mutagenesis screen to identify CYP-36A1 effectors that regulate egg-laying behavior, stress responses, and gene expression. We screened for mutations that suppressed both the low *P_T24B8.5_::gfp* expression and normal egg laying of *cyp-36A1(lf); egl-9(lf)* double mutants, looking for triple mutants that, like *egl-9(lf)* single mutants, had high GFP expression and were egg-laying defective. By screening for suppressors of the two abnormalities simultaneously, we were able to focus on effectors of CYP-36A1 rather than finding genes that affect only egg laying or only expression of *T24B8.5* independently of the EGL-9/HIF-1/CYP-36A1 pathway. From this screen we identified one nonsense and one missense allele of the nuclear receptor gene *nhr-46* (Figure 4A–4D and Figure 4—figure supplement 1), both of which caused an egg-laying defect and high expression of the *P_T24B8.5_::gfp* reporter. We tested whether *nhr-46* also functions in regulating stress responses downstream of *cyp-36A1* and found that *cyp-36A1(lf); nhr-46(lf); egl-9(lf)* triple mutants were more resistant to *Pseudomonas* infection, ER stress, and oxidative stress than *cyp-36A1(lf); egl-9(lf)* double mutants (Figure 4E–4G and Figure 4—figure supplement 2). Thus, NHR-46 is a downstream effector of CYP-36A1 in regulation of stress resistance as well as of behavior and gene expression. Interestingly, *nhr-46(lf)* single mutants displayed wild-type egg laying (Figure 4D), tunicamycin resistance (Figure 4F), and oxidative stress resistance (Figure 4G), and nearly wild-type survival on *Pseudomonas* (Figure 4E and Figure 4—figure supplement 2), indicating that in addition to *nhr-46* at least one other pathway is required to transduce *egl-9*-mediated modulation of egg laying and stress resistance. For example, in *egl-9(lf)* mutants or hypoxia, HIF-1 might drive expression of two (or more) targets that act together to inhibit egg laying and promote stress resistance. Thus, *cyp-36A1* activation and consequent *nhr-46* inhibition would promote egg-laying inhibition and stress resistance in *egl-9(lf)* mutant animals – in which this second pathway was also activated – but not in *nhr-46(lf)* single mutants – in which this second pathway was not activated. *nhr-46(lf)* single mutants had increased expression of the *P_T24B8.5_::gfp* reporter (Figure 4B and C and Figure 4—figure supplement 1), suggesting that at least some gene expression changes can be mediated by *nhr-46* alone.

**Figure 4—figure supplement 1.**
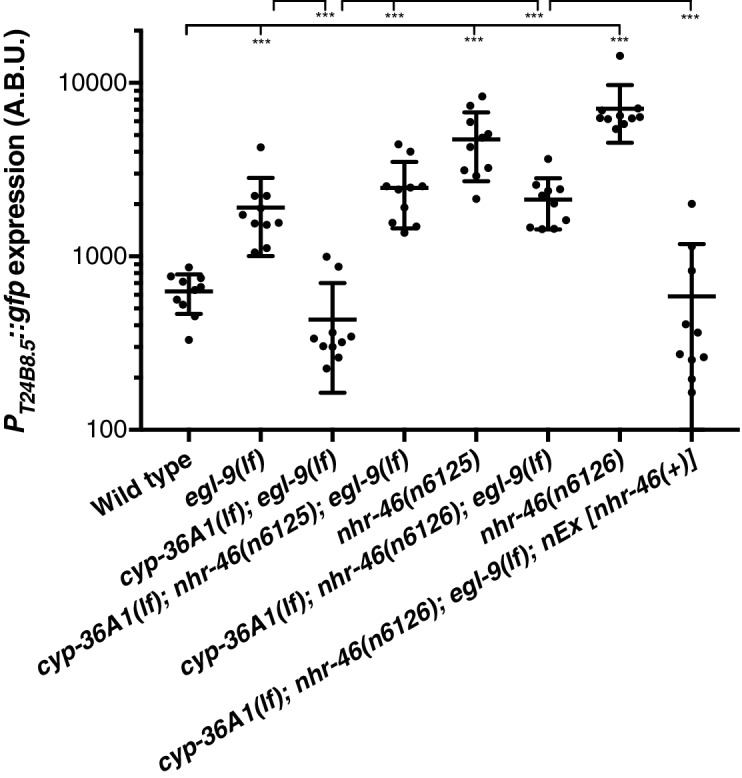
Replicate data for *P_T24B8.5_::gfp* reporter expression. Replicate data for Figure 4C. Quantification of *n* = 10 animals. ***p<0.001 considered significant (Student’s t-test with Holm-Bonferroni correction).

**Figure 4—figure supplement 2.**
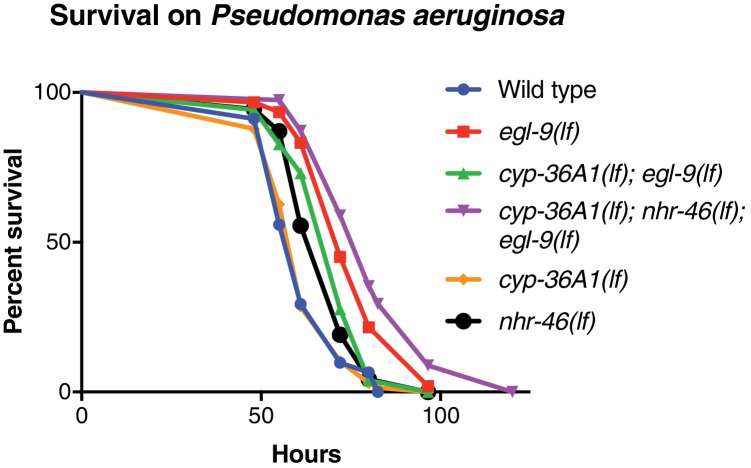
Replicate data for survival on *Pseudomonas aeruginosa*. Replicate data for Figure 4E. Wild type (*n* = 34) vs. *egl-9(n586)* (*n* = 61), p<0.001; *egl-9(lf)* vs. *cyp-36A1(lf); egl-9(lf)* (*n* = 52), p<0.05; *cyp-36A1(lf); egl-9(lf)* vs. *cyp-36A1(lf); nhr-46(lf); egl-9(lf)* (*n* = 39), p<0.001; wild type vs. *cyp-36A1(lf)* (*n* = 91), p>0.05; wild type vs. *nhr-46(lf)* (*n* = 54), p<0.05, as determined by the log-rank (Mantel-Cox) test, correcting for multiple comparisons with the Holm-Bonferroni method.

### *nhr-46* functions tissue-specifically to regulate gene expression and behavior

**Figure 5.**
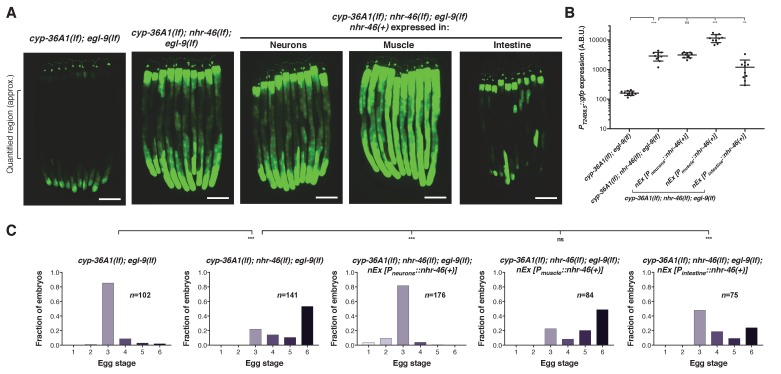
NHR-46 acts in different tissues to regulate *T24B8.5* expression and egg laying. (**A**) Expression of *nhr-46(+)* in the intestine but not in neurons or muscle rescued the high *P_T24B8.5_::gfp* expression in the intestine of *cyp-36A1(lf); nhr-46(lf); egl-9(lf)* mutants (*n* = 10 animals per image). Scale bars, 100 μm. (**B**) Quantification of fluorescence intensity for (**A**), measured as average intensity for a 300 μm section of the intestine in the midbody of each animal, as indicated. **p<0.01, ***p<0.001 considered significant. ns (p>0.05), not significant (Student’s t-test with Holm-Bonferroni correction). Mean ±SD of *n* = 10 animals. See figure supplement for replicate data. (**C**) Distribution of stages of eggs laid by adult hermaphrodites. Expression of *nhr-46(+)* in the intestine partially rescued the egg-laying defect of *cyp-36A1(lf); nhr-46(lf); egl-9(lf)* mutants. Neuronal *nhr-46(+)* expression fully rescued the egg-laying defect. Expression of *nhr-46(+)* in muscle did not rescue the egg-laying defect of *cyp-36A1(lf); nhr-46(lf); egl-9(lf)* mutants. ***p<0.001 considered significant. ns (p>0.05), not significant (Chi-square test with Holm-Bonferroni correction). Alleles used were *egl-9(sa307), cyp-36A1(gk824636)*, *nhr-46(n6126)*, *nEx2713 [P_neurons_::nhr-46(+)]*, *nEx2715 [P_muscle_::nhr-46(+)]*, and *nEx2864 [P_intestine_::nhr-46(+)].* All strains contained the *agIs219* (*P_T24B8.5_::gfp*) transgene.

*nhr-46* is expressed in many tissues, including neurons, hypoderm, muscle, intestine, and the spermatheca (Feng et al., 2012). Tissue-specific expression of *nhr-46* in the intestine, but not in neurons or muscle, rescued the high GFP expression caused by *nhr-46(lf)*, indicating that *nhr-46* acts cell autonomously in the intestine to control intestinal expression of *T24B8.5* (Figure 5A and B and Figure 5—figure supplement 1). *nhr-46* expression in neurons fully rescued the egg-laying defect of *cyp-36A1(lf); nhr-46(lf); egl-9(lf)* triple mutants (Figure 5C), demonstrating that *nhr-46* function in neurons is sufficient to regulate egg-laying behavior. *nhr-46* expression in intestine, but not muscle, also partially rescued the egg-laying defect of *cyp-36A1(lf); nhr-46(lf); egl-9(lf)* triple mutants (Figure 5C). In combination with the tissue-specific CYP-36A1 and HIF-1 experiments described above, these results suggest that a CYP-36A1-regulated cell non-autonomous signal from any tissue can act on NHR-46 in the intestine to drive intestinal *T24B8.5* expression and in either the nervous system or the intestine to regulate egg-laying behavior. That *nhr-46* can act in either of two different tissues to regulate egg laying suggests that a second intercellular signal, for example a peptide or other hormone, might regulate egg laying downstream of NHR-46.

**Figure 5—figure supplement 1.**
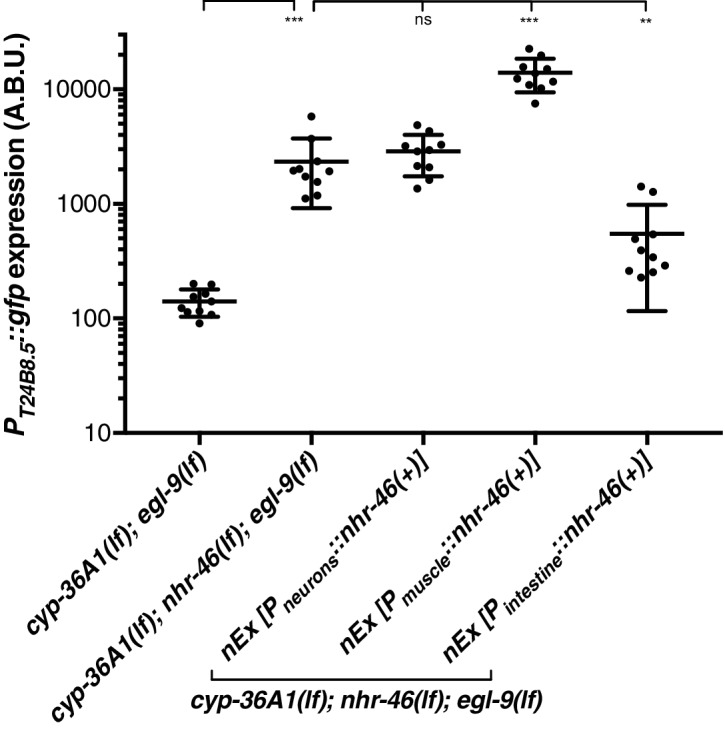
Replicate data for *P_T24B8.5_::gfp* reporter expression. Replicate data for Figure 5B. Quantification of *n* = 10 animals. **p<0.01, ***p<0.001 considered significant. ns (p>0.05), not significant (Student’s t-test with Holm-Bonferroni correction).

## Discussion

**Figure 6.**
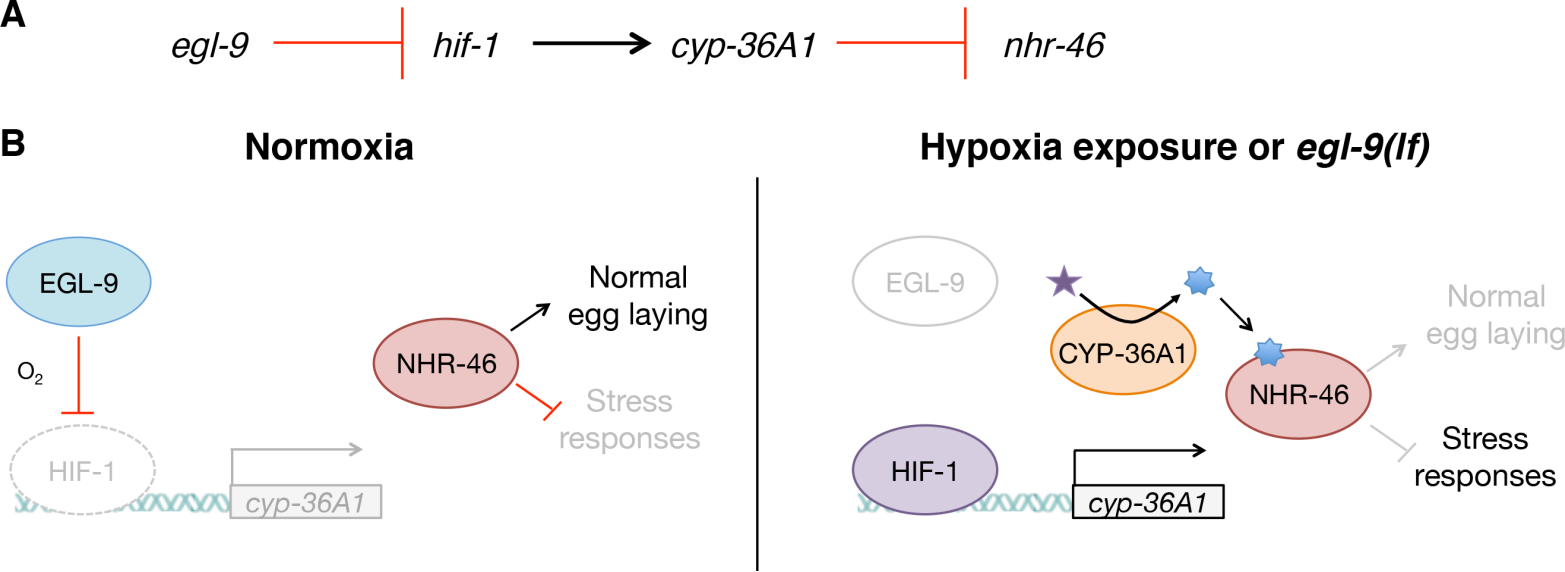
Model for the regulation of physiology and behavior by NHR-46 and CYP-36A1. (**A**) The genetic pathway in which *egl-9* inhibits *hif-1*, which activates *cyp-36A1*, which in turn inhibits *nhr-46*. (**B**) Model for how CYP-36A1 and NHR-46 function downstream of HIF-1. We suggest that CYP-36A1, which is transcriptionally upregulated by HIF-1, generates a hormone that binds NHR-46, thereby promoting transcriptional and physiological changes. See text for details.

These studies define a novel molecular genetic pathway that mediates cell non-autonomous regulation of gene expression by the HIF-1 transcription factor. Our genetic analysis indicates that *hif-1* activates the cytochrome P450 *cyp-36A1*, which in turn inhibits the nuclear receptor *nhr-46* (Figure 6A). We speculate that the molecular function of CYP-36A1 is to generate an unidentified hormone that binds and regulates NHR-46, similar to other cytochrome P450 enzymes that function upstream of nuclear receptors (Evans and Mangelsdorf, 2014) and consistent with our observed cell non-autonomous function of CYP-36A1. We propose the following model (Figure 6B): In wild-type animals, EGL-9 inhibits HIF-1 activity, such that the HIF-1 target *cyp-36A1* is not transcribed. The unliganded NHR-46 represses expression of genes that promote stress resistance and inhibit egg laying. In *egl-9(lf)* mutants, as in hypoxia-exposed worms, HIF-1 is stabilized and drives increased *cyp-36A1* expression. A CYP-36A1-generated hormone then binds NHR-46 and antagonizes the repressive function of NHR-46, accounting for the observed negative regulatory relationship between *cyp-36A1* and *nhr-46*. Ligand-bound NHR-46 is likely activated to promote the expression of target genes, by analogy to a well-established mechanism of nuclear receptor regulation in which ligand binding mediates a switch from repressive to activating nuclear receptor function (Evans and Mangelsdorf, 2014). Alternatively, CYP-36A1 might degrade a ligand that activates NHR-46.

### Cell non-autonomous regulation of stress resistance by HIF involves multiple pathways

Numerous studies have reported that HIF-1 promotes longevity and stress resistance of *C. elegans* (Darby et al., 1999; Treinin et al., 2003; Bellier et al., 2009; Mehta et al., 2009; Zhang et al., 2009; Chen et al., 2009; Lee et al., 2010; Shao et al., 2010; Budde and Roth, 2011; Kirienko et al., 2013; Fawcett et al., 2015). Nonetheless, despite substantial interest in the role of this pathway in stress biology, few relevant HIF effectors have been identified. Interestingly, a recent study reported that HIF-dependent serotonin signaling from the nervous system cell non-autonomously drives expression of the xenobiotic detoxification enzyme flavin-containing monooxygenase-2 (FMO-2) in the intestine, resulting in increased stress resistance and consequent extension of lifespan (Leiser et al., 2015). Here we report that a different signal, likely a lipophilic hormone, acts cell non-autonomously downstream of HIF to regulate gene expression and stress resistance. Consistent with the findings of Leiser et al. (2015), our RNA-seq data showed that in *egl-9(lf)* mutants there is a strong induction of *fmo-2* expression and that this induction is suppressed by *hif-1* mutation (Supplementary file 2), that is HIF-1 upregulates *fmo-2* expression. Notably, this HIF-dependent expression of *fmo-2* does not require *cyp-36A1*: the high *fmo-2* expression of *egl-9(lf)* mutants is not suppressed by *cyp-36A1(lf).* We therefore suggest that serotonin-mediated *fmo-2* expression and *cyp-36A1/nhr-46*-mediated gene expression are parallel pathways downstream of HIF that regulate stress resistance.

### Human cytochrome P450 enzymes might act as mediators of HIF-dependent gene expression

We speculate that some human CYPs might serve as mediators of HIF-dependent gene expression changes through a mechanism analogous to that we describe above for CYP-36A1. In support of this hypothesis, previous studies have identified several human cytochrome P450 enzymes that are putative direct HIF targets based on whole-genome ChIP-chip and ChIP-seq analysis (Mole et al., 2009; Schödel et al., 2011). We note that the humoral nature of a CYP-generated molecule would make it a candidate mediator of non-autonomous regulation of hypoxia response by the EGLN/HIF pathway, such as is observed in remote ischemic preconditioning (Cai et al., 2013; Olenchock et al., 2016).

### Cytochrome P450 enzymes might be major players in the hypoxia-response pathway

We previously identified another cytochrome P450 gene, *cyp-13A12*, as acting downstream of *egl-9* in a locomotory behavior (Ma et al., 2013). In contrast to CYP-36A1, which is upregulated by HIF-1, CYP-13A12 is downregulated by HIF-1 upon hypoxia exposure or in *egl-9(lf)* mutants. The downstream effectors of CYP-36A1 and CYP-13A12 are also distinct, as we show here that CYP-36A1 regulates a nuclear receptor that controls transcription, whereas CYP-13A12 generates eicosanoids that act on a seconds-to-minutes timescale unlikely to require gene expression changes. Thus, different cytochrome P450 enzymes can act broadly, through multiple mechanisms, downstream of the EGL-9/HIF-1 hypoxia-response pathway in *C. elegans.* We propose that cytochrome P450 enzymes might similarly be important HIF effectors in mammals. Polymorphisms in numerous human cytochrome P450 genes have been associated with cardiovascular disease (Elbekai and El-Kadi, 2006; Rowland and Mangoni, 2014), for which HIF plays a protective role (Semenza, 2012), and with cancers (Agundez, 2004), for which HIF contributes to pathogenesis (Semenza, 2012). Furthermore, a study of genetic adaptations in humans to the environmentally hypoxic Tibetan plateau identified well-established members of the HIF pathway and, intriguingly, also noted positive selection at two cytochrome P450 loci (Simonson et al., 2010). Together these observations suggest that the cytochrome P450 family of enzymes is important in a wide range of hypoxia-associated contexts in humans. We suggest the presence of a mechanistic link between the canonical HIF pathway and the function of cytochrome P450 enzymes in humans and posit that an understanding of how these highly druggable enzymes (Schuster and Bernhardt, 2007) control processes downstream of HIF might reveal new therapeutic avenues for treating a broad array of disorders.

Cytochrome P450 enzymes require oxygen as a substrate and thus might be regulated by oxygen availability. A long-standing issue in the understanding of HIF function is ‘range finding,’ that is how activity of the EGLN/HIF pathway is modulated such that it responds to different oxygen set points depending on context to drive a diversity of biological outputs (Ratcliffe, 2013). We hypothesize that the oxygen sensitivity of HIF-regulated cytochrome P450 enzymes might enable them to function in such a range finding mechanism to narrow the range of oxygen concentrations at which a subset of HIF-regulated physiological changes are activated.

## Materials and methods

**Key resources table:** 

Reagent type (species) or resource	Designation	Source or reference	Identifiers	Additional information
Strain, strain background (*Caenorhabditis elegans*)	AU78	Dennis Kim		*agIs9 III*
Strain, strain background (*C. elegans*)	CB6088	Jonathan Hodgkin		*egl-9(sa307) V hif-1(ia4) V*
Strain, strain background (*C. elegans*)	JT307	Creg Darby		*egl-9(sa307) V*
Strain, strain background (*C. elegans*)	MT20483	Dengke Ma/Bob Horvitz		*nIs470 IV*
Strain, strain background (*C. elegans*)	MT22836	this paper		*cyp-36A1(gk824636) I ; egl-9(sa307) V*
Strain, strain background (*C. elegans*)	MT23218	this paper		*nIs682 X*
Strain, strain background (*C. elegans*)	MT24164	this paper		*cyp-36A1(gk824636) I ; agIs9 III*
Strain, strain background (*C. elegans*)	MT24165	this paper		*cyp-36A1(gk824636) I ; agIs9 III ;* *egl-9(sa307) V*
Strain, strain background (*C. elegans*)	MT24166	this paper		*agIs9 III ; egl-9(sa307) V hif-1(ia4) V*
Strain, strain background (*C. elegans*)	MT24167	this paper		*agIs9 III ; egl-9(sa307) V*
Strain, strain background (*C. elegans*)	MT24177	this paper		*agIs9 III ; hif-1(ia4) V*
Strain, strain background (*C. elegans*)	MT24179	this paper		*cyp-36A1(n5666) I ; (nIs470) IV*
Strain, strain background (*C. elegans*)	MT24520	this paper		*cyp-36A1(n5666) I ; (nIs470) IV ;* *egl-9(n586ts) V ; nIs674*
Strain, strain background (*C. elegans*)	MT24622	this paper		*cyp-36A1(n5666) I ; (nIs470) IV ;* *egl-9(n586ts) V*
Strain, strain background (*C. elegans*)	MT24684	this paper		*cyp-36A1(gk824636) I ; agIs9 III ;* *nhr-46(n6126) IV ; egl-9(sa307) V ;* *nEx2586*
Strain, strain background (*C. elegans*)	MT24690	this paper		*agIs9 III ; otEx3165*
Strain, strain background (*C. elegans*)	MT24692	this paper		*agIs9 III ; otEx3156*
Strain, strain background (*C. elegans*)	MT24911	this paper		*cyp-36A1(gk824636) I ; agIs9 III ;* *nhr-46(n6126) IV ; egl-9(sa307) V*
Strain, strain background (*C. elegans*)	MT25047	this paper		*agIs9 III ; nEx2699*
Strain, strain background (*C. elegans*)	MT25048	this paper		*cyp-36A1(gk824636) I ; agIs9 III ;* *nEx2699*
Strain, strain background (*C. elegans*)	MT25050	this paper		*cyp-36A1(gk824636) I ; agIs9 III ;* *otEx3156*
Strain, strain background (*C. elegans*)	MT25051	this paper		*cyp-36A1(gk824636) I ; agIs9 III ;* *otEx3165*
Strain, strain background (*C. elegans*)	MT25104	this paper		*cyp-36A1(gk824636) I ; agIs9 III ;* *nhr-46(n6126) IV ; egl-9(sa307) V ;* *nEx2713*
Strain, strain background (*C. elegans*)	MT25107	this paper		*cyp-36A1(gk824636) I ; agIs9 III ;* *nhr-46(n6126) IV ; egl-9(sa307) V ;* *nEx2715*
Strain, strain background (*C. elegans*)	MT25196	this paper		*agIs9 III ; nhr-46(n6125) IV*
Strain, strain background (*C. elegans*)	MT25197	this paper		*cyp-36A1(gk824636) I ; agIs9 III ;* *nhr-46(n6125) IV ; egl-9(sa307) V*
Strain, strain background (*C. elegans*)	MT25211	this paper		*cyp-36A1(gk824636) I*
Strain, strain background (*C. elegans*)	MT25212	this paper		*cyp-36A1(gk824636) I ; egl-9(n586ts) V*
Strain, strain background (*C. elegans*)	MT25213	this paper		*cyp-36A1(gk824636) I ;* *nhr-46(n6126) IV ; egl-9(n586ts) V*
Strain, strain background (*C. elegans*)	MT25214	this paper		*nhr-46(n6126) IV*
Strain, strain background (*C. elegans*)	MT25215	this paper		*egl-9(n586ts) V*
Strain, strain background (*C. elegans*)	MT25596	this paper		*cyp-36A1(gk824636) I ; agIs9 III ;* *egl-9(sa307) V ; nEx2853*
Strain, strain background (*C. elegans*)	MT25599	this paper		*cyp-36A1(gk824636) I ; agIs9 III ;* *egl-9(sa307) V ; nEx2856*
Strain, strain background (*C. elegans*)	MT25601	this paper		*cyp-36A1(gk824636) I ; agIs9 III ;* *egl-9(sa307) V ; nEx2849*
Strain, strain background (*C. elegans*)	MT25605	this paper		*cyp-36A1(gk824636) I ; agIs9 III ;* *egl-9(sa307) V ; nEx2859*
Strain, strain background (*C. elegans*)	MT25606	this paper		*agIs9 III ; nEx2860*
Strain, strain background (*C. elegans*)	MT25610	this paper		*cyp-36A1(gk824636) I ; agIs9 III ;* *nhr-46(n6126) IV ; egl-9(sa307) V ; nEx2864*
Strain, strain background (*C. elegans*)	MT25611	this paper		*cyp-36A1(gk824636) I ; agIs9 III ; nEx2860*
Strain, strain background (*C. elegans*)	MT25627	this paper		*agIs9 III ; nhr-46(n6126) IV*
Strain, strain background (*C. elegans*)	ZG31	Huaqi Jiang/Jo Anne Powell-Coffman		*hif-1(ia4) V*

### *C. elegans* strains and transgenes

All *C. elegans* strains were cultured as described previously (Brenner, 1974). We used the N2 Bristol strain as the reference wild-type strain, and the polymorphic Hawaiian strain CB4856 (Davis et al., 2005) for genetic mapping and SNP analysis. We used the following mutations and transgenes:

LGI: *cyp-36A1(n5666, gk824636)*

LGIII: *agIs219[P_T24B8.5_::gfp::unc-54 3’UTR, P_ttx-3_::gfp::unc-54 3’UTR]*

LGIV: *nhr-46(n6125, n6126), nIs470[P_cysl-2_::gfp, P_myo-2_::mCherry]*, *him-8(e1489)*

LGV: *egl-9(n586, sa307)*, *hif-1(ia4)*

LGX: *nIs682[P_cyp-36A1_::gfp::unc-54 3’UTR, P_myo-3_::mCherry::unc-54 3’UTR]*

Unknown linkage: *nIs674[P_cyp-36A1_::cyp-36A1(+) gDNA::cyp-36A1 3’UTR, P_myo-3_::mCherry::unc-54 3’UTR]*

### Extrachromosomal arrays

o*tEx3156 [P_dpy-7_::hif-1(P621A), P_ttx-3_::rfp]*, *otEx3165 [P_unc-120_::hif-1(P621A), P_ttx-3_::rfp]*, *nEx2699 [P_rab-3_::hif-1(P621A)::F2A::mCherry::tbb-2 3'UTR, P_ttx-3_::mCherry]*, *nEx2860 [P_vha-6_::hif-1(P621A)::F2A::mCherry::tbb-2 3'UTR, rol-6(su1006dm)], nEx2849 [P_vha-6_::cyp-36A1 cDNA::F2A::mCherry::tbb-2 3'UTR, rol-6(su1006dm)], nEx2856 [P_dpy-7_::cyp-36A1 cDNA::F2A::mCherry::tbb-2 3'UTR, rol-6(su1006dm)], nEx2853 [P_rab-3_::cyp-36A1 cDNA::F2A::mCherry::tbb-2 3'UTR, rol-6(su1006dm)], nEx2859 [P_unc-54_::cyp-36A1 cDNA::F2A::mCherry::tbb-2 3'UTR, P_ttx-3_::mCherry::tbb-2 3’UTR], nEx2586 [P_nhr-46_::nhr-46(+) gDNA::nhr-46 3’UTR, P_myo-3_::mCherry::unc-54 3’UTR], nEx2715[P_unc-54_::nhr-46 cDNA::F2A::mCherry::tbb-2 3'UTR]*, *nEx2713 [P_rab-3_::nhr-46 cDNA::F2A::mCherry::tbb-2 3'UTR]*, *nEx2864 [P_vha-6_::nhr-46 cDNA::F2A::mCherry::tbb-2 3'UTR, rol-6(su1006dm)]*

Note on allele usage: For *egl-9*, the weaker *n586* allele was used for the screen and in the initial phenotypic characterization of screen mutants (Figure 1A–1F and Figure 2C–2E). The stronger *sa307* allele was used for all other experiments, except for in the slow killing assay, for which the *sa307* allele is less protective than weaker alleles, as previously reported (Bellier et al., 2009). For *cyp-36A1*, the allele identified from the screen, *n5666*, was used in the initial phenotypic characterization (Figure 1D–1F and 2C–2E); the putative null allele *gk824636* was used for all other experiments. Alleles for other genes were used as indicated in the figure legends.

### Molecular biology and transgenic strain construction

The *P_cyp-36A1_::gfp::unc-54 3’UTR* construct (transgene *nIs682)* was generated by using PCR fusion (Hobert, 2002) to fuse a PCR product containing the *cyp-36A1* promoter fragment (4.4 kb of upstream sequence) to a PCR product containing *gfp::unc-54 3’UTR.* The *cyp-36A1* rescuing construct (transgene *nIs674*) was generated by amplifying a PCR product from gDNA containing 4.4 kb upstream, the *cyp-36A1* locus, and 1.6 kb downstream. The *nhr-46* rescuing construct (transgene *nEx2586*) was generated by amplifying a PCR product from gDNA containing 1.9 kb upstream, the *nhr-46* locus, and 0.9 kb downstream. All remaining constructs were generated using the Infusion cloning technique (Clontech). The promoter fragments used for *dpy-7* (hypoderm) (Gilleard et al., 1997), *vha-6* (intestine) (Allman et al., 2009) *rab-3* (neurons) (Mahoney et al., 2006) and *unc-54* (muscle) (D. Ma, personal communication) contain 1.3, 0.9, 1.4, and 1.9 kb, respectively, of sequence upstream of the start codons of each of these genes. Expression of tissue-specific rescuing transgenes was confirmed using mCherry tagging. We noticed expression in some unidentified cells outside the body-wall muscle near the junction of the pharynx and intestine in the strain *cyp-36A1(gk824636); agIs9; egl-9(sa307); nEx2859 [P_unc-54_::cyp-36A1 cDNA::F2A::mCherry::tbb-2 3'UTR, P_ttx-3_::mCherry::tbb-2 3’UTR]*; such expression might contribute to rescue of the mutant phenotype in that strain. *C45E5.6b* was used for *nhr-46* cDNA. *F38A6.3a* with a P621A stabilizing mutation was used for *hif-1* cDNA (Pocock and Hobert, 2008). Where present, the F2A sequence served as a ribosomal skip sequence to cause separation of the two peptides encoded before and after the F2A (Ahier and Jarriault, 2014). Transgenic strains were generated by germline transformation as described (Mello et al., 1991). All transgenic constructs were injected at 2.5–50 ng/μl.

### Mutagenesis screen for suppressors of *egl-9*

To screen for suppressors of the *egl-9* egg-laying defect, we mutagenized *egl-9(n586)* mutants with ethyl methanesulfonate (EMS) as described previously (Brenner, 1974). The starting strain contained the *P_cysl-2_::gfp (nIs470)* transgene, which is highly expressed in *egl-9(lf)* mutants and served as a reporter for HIF-1 activity (Ma et al., 2012). We used a dissecting microscope to screen the F2 progeny for suppression of the egg-laying defect (i.e. the Egl phenotype), picking (1) adults that appeared less Egl than *egl-9(n586)* mutants, and (2) eggs laid by the F2 animals that were at an earlier developmental stage than those laid by *egl-9(n586)* mutants. Screen isolates were backcrossed to determine dominant vs. recessive and single-gene inheritance pattern and crossed to *him-8(e1489); egl-9(sa307) hif-1(ia4)* to test complementation with *hif-1(lf)*. The screen allele *n5666*, which conferred a recessive phenotype and was not allelic to *hif-1*, mapped between SNPs *pkP1052* and *rs3139013* on LGI with SNP mapping (Davis et al., 2005) using a strain containing *egl-9(n586)* introgressed into the Hawaiian strain CB4856 (Ma et al., 2013). Whole-genome sequencing identified a mutation in *cyp-36A1* in the *n5666* interval, and transgenic rescue demonstrated that this *cyp-36A1* mutation is the causative mutation, as described in the text.

### Mutagenesis screen for suppressors of *cyp-36A1*

To screen for downstream effectors of *cyp-36A1*, we mutagenized *cyp-36A1(gk824636); egl-9(sa307)* with ethyl methanesulfonate (EMS). The starting strain contained the *P_T24B8.5_::gfp (agIs219)* transgene, which has low expression in *cyp-36A1(gk824636); egl-9(sa307)* mutants and served as a reporter for CYP-36A1 activity. We used a dissecting microscope equipped to examine GFP fluorescence to screen for F2 progeny with high GFP fluorescence and an Egl appearance. The only two isolates failed to complement and were found to be alleles of *nhr-46* by whole-genome sequencing and transgenic rescue. The mutant phenotypes of *cyp-36A1(lf); n6126; egl-9(lf)* were rescued by an *nhr-46(+)* transgene, demonstrating that the mutation in *nhr-46* is the causative mutation and suggesting that *n6126* is a loss-of-function allele.

### Behavioral assays

To quantify egg-laying behavior, we scored the developmental stages of eggs laid by young adult hermaphrodites as described previously (Ringstad and Horvitz, 2008). Egg-laying defective mutants retain eggs longer in the uterus, thus laying them at later developmental stages. To examine egg-laying behavior after exposure to hypoxia, young adult animals were placed in a hypoxia chamber (Coy Laboratory) at 1% O_2_ balanced by N_2_ for 24 hr, after which the egg-laying assay was performed in normoxia. Control animals were exposed to room air (21% O_2_) for 24 hr; animals were randomly allocated to 1% or 21% O_2_ treatment. Locomotion assays were performed on bacterial food and quantified using a custom worm tracker, as described previously (Paquin et al., 2016). Defecation assays were performed as described previously (Thomas, 1990), counting the number of defecation cycles in ten minutes.

### *Pseudomonas aeruginosa* killing assay

Sensitivity to the *Pseudomonas aeruginosa* strain PA14 was assayed using the big lawn killing assay as described previously (Reddy et al., 2009). The big lawn killing assay was used to remove any influence of avoidance behavior on survival, as wild-type PA14 avoidance is dependent on normal aerotaxis behavior (Reddy et al., 2009), and *egl-9(lf)* mutants have previously been shown to display abnormal aerotaxis (Chang and Bargmann, 2008).

### Tunicamycin survival assay

Sensitivity to tunicamycin was assayed by placing at least 100 starvation-synchronized L1 animals on NGM plates containing 5 μg/ml tunicamycin (Sigma), made using 10 mg/ml tunicamycin stock in DMSO and seeded with *E. coli* OP50 bacteria. Survival to the L4 larval stage or later was determined after three days.

### t-BOOH survival assay

Sensitivity to tert-butyl hydroperoxide (t-BOOH) was assayed by placing ~60 young adult worms on NGM plates containing 7.5 mM t-BOOH, made using 70% t-BOOH solution (Sigma) and seeded with *E. coli* OP50 bacteria. Survival was evaluated after 10 hr.

### Microscopy

Epifluorescence images of *P_T4B8.5_::gfp* expression were obtained using an AxioImager Z2 upright microscope (Zeiss) and ZEN software (Zeiss). Confocal images of *P_cyp-36A1_::gfp* expression were obtained using an LSM 800 instrument (Zeiss) and ZEN software. Fluorescence intensity for the *P_T24B8.5_::gfp* reporter was quantified by measuring average intensity in a 300 μm section of the intestine centered on the vulva using FIJI software. *P_T24B8.5_::gfp* reporter imaging conditions were optimized for observation of fluorescence in the midbody; fluorescence was not saturated in this region in quantified images.

### RNA isolation for qRT-PCR and RNA-seq

All strains were maintained at 22.5**°**C for at least two generations without starvation prior to experiment. ~150 very young adults (0–1 eggs in uterus) were picked into M9 buffer and allowed to settle. M9 was aspirated, and worms were then rinsed twice with M9 and twice with RNase-free water. Excess liquid was aspirated and the pellet was frozen in liquid nitrogen. RLT buffer (QIAGEN) was added to the frozen pellet, and worms were lysed using a BeadBug microtube homogenizer (Sigma) and 0.5 mm zirconium beads (Sigma). RNA was extracted using the RNeasy Mini kit (QIAGEN) according to the manufacturer’s instructions.

### *cyp-36A1* mRNA expression analysis by qRT-PCR

Reverse transcription was performed using SuperScript III (Invitrogen). Quantitative PCR was performed using Applied Biosystems Real-Time PCR Instruments. Expression levels were normalized to the expression of the ribosomal subunit gene *rpl-32*.

### Primers for qRT-PCR

*cyp-36A1* F: ACCAGCTTGTCCAACACCAA

*cyp-36A1* R: CACGCTTTGGCTCCCATTTC

*rpl-32* F: GGCTACACGACGGTATCTGT

*rpl-32* R: CAAGGTCGTCAAGAAGAAGC

### RNA-seq library preparation

RNA integrity and concentration were checked on a Fragment Analyzer (Advanced Analytical). The mRNA was purified by polyA-tail enrichment, fragmented, and reverse transcribed into cDNA (Illumina TruSeq). cDNA samples were then end-repaired and adaptor-ligated using the SPRI-works Fragment Library System I (Beckman Coulter Genomics) and indexed during amplification. Libraries were quantified using the Fragment Analyzer (Advanced Analytical) and qPCR before being loaded for single-end sequencing using the Illumina HiSeq 2000.

### RNA-seq data analysis

Reads were aligned against the *C. elegans* ce10 genome assembly using bwa 0.7.5a (Li and Durbin, 2009) and samtools/0.1.19 (1000 Genome Project Data Processing Subgroup et al., 2009) (bwa aln/bwa samse), and mapping rates, fraction of multiply-mapping reads, number of unique 20-mers at the 5’ end of the reads, insert size distributions and fraction of ribosomal RNAs were calculated using dedicated perl scripts and bedtools v. 2.17.0 (Quinlan and Hall, 2010). For expression analysis, reads were aligned against the *C. elegans* ce10 genome/ENSEMBL 65 annotation using RSEM 1.2.15 (Li and Dewey, 2011) and bowtie 1.0.1 (Langmead et al., 2009), with the following parameters: -p 6 --bowtie-chunkmbs 1024 --output-genome-bam. Raw expected read counts were retrieved and used for differential expression analysis with Bioconductor’s edgeR package in the R 3.2.3 statistical environment (Robinson et al., 2010). First, common, trended, and gene-specific read dispersion across sequencing libraries and genes was estimated using the estimateDisp function. Given the small number of replicates, a gene-wise negative binomial generalized linear model (GLM) with quasi-likelihood tests (as implemented in the glmQLFit function) was used to test for differential expression between conditions (Lun et al., 2016). Briefly, this statistical framework works by first fitting the observed and expected distributions of read counts for each gene across conditions using a GLM, which is based on the negative binomial distribution and the observed read dispersion. The significance of biases in read counts is then tested using the quasi-likelihood F-test (implemented in glmQLFTest). This test provides more robust and reliable error rate control at low number of replicates, because it reflects the uncertainty in read distribution better than the likelihood ratio test. Models were fitted across all conditions and relevant differential expression testing was performed using glmQLFTest between pairs of conditions of interest. P values were adjusted for multiple comparisons using the Benjamini-Hochberg procedure (Benjamini and Hochberg, 1995). Gene ontology enrichment analysis was performed using GOrilla (Eden et al., 2009), examining genes that were significantly downregulated in *cyp-36A1(lf); egl-9(lf)* double mutants vs. *egl-9(lf)* single mutants (i.e. orange circle in Figure 2B) as compared to genes that were at least twofold upregulated in *egl-9(lf)* mutants vs. wild type (adjusted p value<0.05) and significantly downregulated in *egl-9(lf) hif-1(lf)* vs. *egl-9(lf)* (adjusted p value<0.05) (i.e. purple circle in Figure 2B).

### Statistical analysis

Chi-square tests were used to compare the distribution of stages of eggs laid by wild-type and mutant animals. Unpaired t-tests were used to compare *cyp-36A1* mRNA expression between strains, survival on tunicamycin between strains, survival on t-BOOH between strains, and *P_T24B8.5_::gfp* reporter fluorescence intensity between strains. Log-rank (Mantel-Cox) tests were used to compare survival of different strains on *Pseudomonas aeruginosa.* In cases of multiple comparisons, a Holm-Bonferroni correction was applied. Statistical tests were performed using GraphPad Prism software (Graphpad Prism, RRID:SCR_002798) version 7.0a. Biological replicates were performed using separate populations of animals.

### Accession numbers

The GEO accession number for the RNA-seq dataset in this paper is GSE108283.

## Additional files

Supplementary file 1. Genes that were significantly differentially expressed between wild type and *egl-9(lf)* and at least twofold downregulated in *egl-9(lf)* vs. wild type.

Supplementary file 2. Genes that were significantly differentially expressed between wild type and *egl-9(lf)* and at least twofold upregulated in *egl-9(lf)* vs. wild type.

Transparent reporting form

### Data availability

Sequencing data have been deposited in GEO under accession code GSE108283.

The following dataset was generated:

Pender CLHorvitz HR2017Hypoxia-inducible factor cell non-autonomously regulates C. elegans stress responses and behavior via a nuclear receptorwww.ncbi.nlm.nih.gov/geo/query/acc.cgi?acc=GSE108283Publicly available at the NCBI Gene Expression Omnibus (accession no: GSE108283).

## References

[bib1] Agundez JA (2004). Cytochrome P450 gene polymorphism and cancer. Current Drug Metabolism.

[bib2] Ahier A, Jarriault S (2014). Simultaneous expression of multiple proteins under a single promoter in *Caenorhabditis elegans* via a versatile 2A-based toolkit. Genetics.

[bib3] Allman E, Johnson D, Nehrke K (2009). Loss of the apical V-ATPase a-subunit VHA-6 prevents acidification of the intestinal lumen during a rhythmic behavior in *C. elegans*. American Journal of Physiology-Cell Physiology.

[bib4] Angeles-Albores D, Puckett Robinson C, Williams BA, Wold BJ, Sternberg PW (2018). Reconstructing a metazoan genetic pathway with transcriptome-wide epistasis measurements. Proceedings of the National Academy of Sciences.

[bib5] Bellier A, Chen CS, Kao CY, Cinar HN, Aroian RV (2009). Hypoxia and the hypoxic response pathway protect against pore-forming toxins in *C. elegans*. PLoS Pathogens.

[bib6] Benjamini Y, Hochberg Y (1995). Controlling the false discovery rate: a practical and powerful approach to multiple testing. Journal of the Royal Statistical Society. Series B.

[bib7] Bishop T, Lau KW, Epstein AC, Kim SK, Jiang M, O'Rourke D, Pugh CW, Gleadle JM, Taylor MS, Hodgkin J, Ratcliffe PJ (2004). Genetic analysis of pathways regulated by the von Hippel-Lindau tumor suppressor in *Caenorhabditis elegans*. PLoS Biology.

[bib8] Brenner S (1974). The genetics of *Caenorhabditis elegans*. Genetics.

[bib9] Budde MW, Roth MB (2011). The response of *Caenorhabditis elegans* to hydrogen sulfide and hydrogen cyanide. Genetics.

[bib10] Cai Z, Luo W, Zhan H, Semenza GL (2013). Hypoxia-inducible factor 1 is required for remote ischemic preconditioning of the heart. PNAS.

[bib11] Chang AJ, Bargmann CI (2008). Hypoxia and the HIF-1 transcriptional pathway reorganize a neuronal circuit for oxygen-dependent behavior in *Caenorhabditis elegans*. PNAS.

[bib12] Chen D, Thomas EL, Kapahi P (2009). HIF-1 modulates dietary restriction-mediated lifespan extension via IRE-1 in *Caenorhabditis elegans*. PLoS Genetics.

[bib13] Darby C, Cosma CL, Thomas JH, Manoil C (1999). Lethal paralysis of *Caenorhabditis elegans* by *Pseudomonas aeruginosa*. PNAS.

[bib14] Davis MW, Hammarlund M, Harrach T, Hullett P, Olsen S, Jorgensen EM (2005). Rapid single nucleotide polymorphism mapping in *C. elegans*. BMC Genomics.

[bib15] Dennis EA, Norris PC (2015). Eicosanoid storm in infection and inflammation. Nature Reviews Immunology.

[bib16] Eden E, Navon R, Steinfeld I, Lipson D, Yakhini Z (2009). GOrilla: a tool for discovery and visualization of enriched GO terms in ranked gene lists. BMC Bioinformatics.

[bib17] Elbekai RH, El-Kadi AO (2006). Cytochrome P450 enzymes: central players in cardiovascular health and disease. Pharmacology & Therapeutics.

[bib18] Epstein AC, Gleadle JM, McNeill LA, Hewitson KS, O'Rourke J, Mole DR, Mukherji M, Metzen E, Wilson MI, Dhanda A, Tian YM, Masson N, Hamilton DL, Jaakkola P, Barstead R, Hodgkin J, Maxwell PH, Pugh CW, Schofield CJ, Ratcliffe PJ (2001). *C. elegans* EGL-9 and mammalian homologs define a family of dioxygenases that regulate HIF by prolyl hydroxylation. Cell.

[bib19] Evans RM, Mangelsdorf DJ (2014). Nuclear receptors, RXR, and the big bang. Cell.

[bib20] Fawcett EM, Hoyt JM, Johnson JK, Miller DL (2015). Hypoxia disrupts proteostasis in *Caenorhabditis elegans*. Aging Cell.

[bib21] Feng H, Craig H, Hope I, Deplancke B, Gheldof N (2012). Expression pattern analysis of regulatory transcription factors in *Caenorhabditis elegans*. Gene Regulatory Networks.

[bib22] Gerisch B, Antebi A (2004). Hormonal signals produced by DAF-9/cytochrome P450 regulate *C. elegans* dauer diapause in response to environmental cues. Development.

[bib23] Gilleard JS, Barry JD, Johnstone IL (1997). cis regulatory requirements for hypodermal cell-specific expression of the *Caenorhabditis elegans* cuticle collagen gene *dpy-7*. Molecular and Cellular Biology.

[bib24] Hobert O (2002). PCR fusion-based approach to create reporter gene constructs for expression analysis in transgenic C. elegans. BioTechniques.

[bib25] Ivan M, Kondo K, Yang H, Kim W, Valiando J, Ohh M, Salic A, Asara JM, Lane WS, Kaelin WG, Jr WGK (2001). HIFalpha targeted for VHL-mediated destruction by proline hydroxylation: implications for O2 sensing. Science.

[bib26] Jaakkola P, Mole DR, Tian YM, Wilson MI, Gielbert J, Gaskell SJ, von Kriegsheim A, Hebestreit HF, Mukherji M, Schofield CJ, Maxwell PH, Pugh CW, Ratcliffe PJ (2001). Targeting of HIF-alpha to the von Hippel-Lindau ubiquitylation complex by O2-regulated prolyl hydroxylation. Science.

[bib27] Jiang H, Guo R, Powell-Coffman JA (2001). The *Caenorhabditis elegans hif-1* gene encodes a bHLH-PAS protein that is required for adaptation to hypoxia. PNAS.

[bib28] Kaelin WG, Ratcliffe PJ (2008). Oxygen sensing by metazoans: the central role of the HIF hydroxylase pathway. Molecular Cell.

[bib29] Kirienko NV, Kirienko DR, Larkins-Ford J, Wählby C, Ruvkun G, Ausubel FM (2013). *Pseudomonas aeruginosa* disrupts *Caenorhabditis elegans* iron homeostasis, causing a hypoxic response and death. Cell Host & Microbe.

[bib30] Kudron MM, Victorsen A, Gevirtzman L, Hillier LW, Fisher WW, Vafeados D, Kirkey M, Hammonds AS, Gersch J, Ammouri H, Wall ML, Moran J, Steffen D, Szynkarek M, Seabrook-Sturgis S, Jameel N, Kadaba M, Patton J, Terrell R, Corson M, Durham TJ, Park S, Samanta S, Han M, Xu J, Yan KK, Celniker SE, White KP, Ma L, Gerstein M, Reinke V, Waterston RH (2018). The ModERN resource: genome-wide binding profiles for hundreds of *Drosophila* and *Caenorhabditis elegans* Transcription Factors. Genetics.

[bib31] Langmead B, Trapnell C, Pop M, Salzberg SL (2009). Ultrafast and memory-efficient alignment of short DNA sequences to the human genome. Genome Biology.

[bib32] Lee SJ, Hwang AB, Kenyon C (2010). Inhibition of respiration extends *C. elegans* life span via reactive oxygen species that increase HIF-1 activity. Current Biology.

[bib33] Leiser SF, Miller H, Rossner R, Fletcher M, Leonard A, Primitivo M, Rintala N, Ramos FJ, Miller DL, Kaeberlein M (2015). Cell nonautonomous activation of flavin-containing monooxygenase promotes longevity and health span. Science.

[bib34] Li B, Dewey CN (2011). RSEM: accurate transcript quantification from RNA-Seq data with or without a reference genome. BMC Bioinformatics.

[bib35] Li H, Durbin R (2009). Fast and accurate short read alignment with Burrows-Wheeler transform. Bioinformatics.

[bib36] Li H, Handsaker B, Wysoker A, Fennell T, Ruan J, Homer N, Marth G, Abecasis G, Durbin R, 1000 Genome Project Data Processing Subgroup (2009). The sequence alignment/Map format and SAMtools. Bioinformatics.

[bib37] Lim Y, Lee D, Kalichamy K, Hong SE, Michalak M, Ahnn J, Kim DH, Lee SK (2014). Sumoylation regulates ER stress response by modulating calreticulin gene expression in XBP-1-dependent mode in *Caenorhabditis elegans*. International Journal of Biochemistry & Cell Biology.

[bib38] Lun ATL, Chen Y, Smyth GK (2016). It’s DE-Licious: A Recipe for Differential Expression Analyses of RNA-Seq Experiments Using Quasi-Likelihood Methods in edgeRStatistical Genomics.

[bib39] Ma DK, Rothe M, Zheng S, Bhatla N, Pender CL, Menzel R, Horvitz HR (2013). Cytochrome P450 drives a HIF-regulated behavioral response to reoxygenation by *C. elegans*. Science.

[bib40] Ma DK, Vozdek R, Bhatla N, Horvitz HR (2012). CYSL-1 interacts with the O_2_-sensing hydroxylase EGL-9 to promote H2S-modulated hypoxia-induced behavioral plasticity in *C. elegans*. Neuron.

[bib41] Mahoney TR, Liu Q, Itoh T, Luo S, Hadwiger G, Vincent R, Wang ZW, Fukuda M, Nonet ML (2006). Regulation of synaptic transmission by RAB-3 and RAB-27 in *Caenorhabditis elegans*. Molecular Biology of the Cell.

[bib42] Maxwell PH, Wiesener MS, Chang GW, Clifford SC, Vaux EC, Cockman ME, Wykoff CC, Pugh CW, Maher ER, Ratcliffe PJ (1999). The tumour suppressor protein VHL targets hypoxia-inducible factors for oxygen-dependent proteolysis. Nature.

[bib43] Mehta R, Steinkraus KA, Sutphin GL, Ramos FJ, Shamieh LS, Huh A, Davis C, Chandler-Brown D, Kaeberlein M (2009). Proteasomal regulation of the hypoxic response modulates aging in *C. elegans*. Science.

[bib44] Mello CC, Kramer JM, Stinchcomb D, Ambros V (1991). Efficient gene transfer in *C. elegans*: extrachromosomal maintenance and integration of transforming sequences. The EMBO Journal.

[bib45] Miller DL, Roth MB (2009). *C. elegans* are protected from lethal hypoxia by an embryonic diapause. Current Biology.

[bib46] Mole DR, Blancher C, Copley RR, Pollard PJ, Gleadle JM, Ragoussis J, Ratcliffe PJ (2009). Genome-wide association of hypoxia-inducible factor (HIF)-1alpha and HIF-2alpha DNA binding with expression profiling of hypoxia-inducible transcripts. Journal of Biological Chemistry.

[bib47] Nakazawa MS, Keith B, Simon MC (2016). Oxygen availability and metabolic adaptations. Nature Reviews Cancer.

[bib48] Nebert DW, Wikvall K, Miller WL (2013). Human cytochromes P450 in health and disease. Philosophical Transactions of the Royal Society B: Biological Sciences.

[bib49] Olenchock BA, Moslehi J, Baik AH, Davidson SM, Williams J, Gibson WJ, Chakraborty AA, Pierce KA, Miller CM, Hanse EA, Kelekar A, Sullivan LB, Wagers AJ, Clish CB, Vander Heiden MG, Kaelin WG (2016). EGLN1 inhibition and rerouting of α-Ketoglutarate suffice for remote ischemic protection. Cell.

[bib50] Palazon A, Goldrath AW, Nizet V, Johnson RS (2014). HIF transcription factors, inflammation, and immunity. Immunity.

[bib51] Paquin N, Murata Y, Froehlich A, Omura DT, Ailion M, Pender CL, Constantine-Paton M, Horvitz HR (2016). The conserved VPS-50 protein functions in dense-core vesicle maturation and acidification and controls animal behavior. Current Biology.

[bib52] Park SK, Tedesco PM, Johnson TE (2009). Oxidative stress and longevity in *Caenorhabditis elegans* as mediated by SKN-1. Aging Cell.

[bib53] Pocock R, Hobert O (2008). Oxygen levels affect axon guidance and neuronal migration in *Caenorhabditis elegans*. Nature Neuroscience.

[bib54] Pocock R, Hobert O (2010). Hypoxia activates a latent circuit for processing gustatory information in *C. elegans*. Nature Neuroscience.

[bib55] Powell-Coffman JA (2010). Hypoxia signaling and resistance in *C. elegans*. Trends in Endocrinology & Metabolism.

[bib56] Prahlad V, Morimoto RI (2009). Integrating the stress response: lessons for neurodegenerative diseases from *C. elegans*. Trends in Cell Biology.

[bib57] Quinlan AR, Hall IM (2010). BEDTools: a flexible suite of utilities for comparing genomic features. Bioinformatics.

[bib58] Ratcliffe PJ (2013). Oxygen sensing and hypoxia signalling pathways in animals: the implications of physiology for cancer. Journal of Physiology.

[bib59] Reddy KC, Andersen EC, Kruglyak L, Kim DH (2009). A polymorphism in *npr-1* is a behavioral determinant of pathogen susceptibility in *C. elegans*. Science.

[bib60] Rendic S, Guengerich FP (2015). Survey of human oxidoreductases and cytochrome P450 enzymes involved in the metabolism of xenobiotic and natural chemicals. Chemical Research in Toxicology.

[bib61] Ringstad N, Horvitz HR (2008). FMRFamide neuropeptides and acetylcholine synergistically inhibit egg-laying by *C. elegans*. Nature Neuroscience.

[bib62] Robinson MD, McCarthy DJ, Smyth GK (2010). edgeR: a Bioconductor package for differential expression analysis of digital gene expression data. Bioinformatics.

[bib63] Rowland A, Mangoni AA (2014). Cytochrome P450 and ischemic heart disease: current concepts and future directions. Expert Opinion on Drug Metabolism & Toxicology.

[bib64] Schito L, Rey S (2018). Cell-Autonomous metabolic reprogramming in hypoxia. Trends in Cell Biology.

[bib65] Schuster I, Bernhardt R (2007). Inhibition of cytochromes P450: existing and new promising therapeutic targets. Drug Metabolism Reviews.

[bib66] Schödel J, Oikonomopoulos S, Ragoussis J, Pugh CW, Ratcliffe PJ, Mole DR (2011). High-resolution genome-wide mapping of HIF-binding sites by ChIP-seq. Blood.

[bib67] Semenza GL (2011). Oxygen sensing, homeostasis, and disease. New England Journal of Medicine.

[bib68] Semenza GL (2012). Hypoxia-inducible factors in physiology and medicine. Cell.

[bib69] Shao Z, Zhang Y, Ye Q, Saldanha JN, Powell-Coffman JA (2010). *C. elegans* SWAN-1 binds to EGL-9 and regulates HIF-1-mediated resistance to the bacterial pathogen *Pseudomonas aeruginosa* PAO1. PLoS Pathogens.

[bib70] Shen C, Nettleton D, Jiang M, Kim SK, Powell-Coffman JA (2005). Roles of the HIF-1 hypoxia-inducible factor during hypoxia response in *Caenorhabditis elegans*. Journal of Biological Chemistry.

[bib71] Shivers RP, Kooistra T, Chu SW, Pagano DJ, Kim DH (2009). Tissue-specific activities of an immune signaling module regulate physiological responses to pathogenic and nutritional bacteria in *C. elegans*. Cell Host & Microbe.

[bib72] Shore DE, Ruvkun G (2013). A cytoprotective perspective on longevity regulation. Trends in Cell Biology.

[bib73] Simonson TS, Yang Y, Huff CD, Yun H, Qin G, Witherspoon DJ, Bai Z, Lorenzo FR, Xing J, Jorde LB, Prchal JT, Ge R (2010). Genetic evidence for high-altitude adaptation in Tibet. Science.

[bib74] Taylor RC, Berendzen KM, Dillin A (2014). Systemic stress signalling: understanding the cell non-autonomous control of proteostasis. Nature Reviews Molecular Cell Biology.

[bib75] Thomas JH (1990). Genetic analysis of defecation in *Caenorhabditis elegans*. Genetics.

[bib76] Thompson O, Edgley M, Strasbourger P, Flibotte S, Ewing B, Adair R, Au V, Chaudhry I, Fernando L, Hutter H, Kieffer A, Lau J, Lee N, Miller A, Raymant G, Shen B, Shendure J, Taylor J, Turner EH, Hillier LW, Moerman DG, Waterston RH (2013). The million mutation project: a new approach to genetics in *Caenorhabditis elegans*. Genome Research.

[bib77] Treinin M, Shliar J, Jiang H, Powell-Coffman JA, Bromberg Z, Horowitz M (2003). HIF-1 is required for heat acclimation in the nematode *Caenorhabditis elegans*. Physiological Genomics.

[bib78] Trent C, Tsuing N, Horvitz HR (1983). Egg-laying defective mutants of the nematode *Caenorhabditis elegans*. Genetics.

[bib79] Wang GL, Jiang BH, Rue EA, Semenza GL (1995). Hypoxia-inducible factor 1 is a basic-helix-loop-helix-PAS heterodimer regulated by cellular O_2_ tension. PNAS.

[bib80] Wouters BG, Koritzinsky M (2008). Hypoxia signalling through mTOR and the unfolded protein response in cancer. Nature Reviews Cancer.

[bib81] Zhang Y, Shao Z, Zhai Z, Shen C, Powell-Coffman JA (2009). The HIF-1 hypoxia-inducible factor modulates lifespan in *C. elegans*. PLoS One.

